# Ultrathin palladium nanosheets with selectively controlled surface facets†
†Electronic supplementary information (ESI) available: Synthetic details of the surfactants and ultrathin PdNSs as well as additional electron microscopic images. See DOI: 10.1039/c8sc00605a


**DOI:** 10.1039/c8sc00605a

**Published:** 2018-04-02

**Authors:** Dongdong Xu, Xiaoli Liu, Hao Lv, Ying Liu, Shulin Zhao, Min Han, Jianchun Bao, Jie He, Ben Liu

**Affiliations:** a Jiangsu Key Laboratory of New Power Batteries , Jiangsu Collaborative Innovation Center of Biomedical Functional Materials , School of Chemistry and Materials Science , Nanjing Normal University , Nanjing , Jiangsu 210023 , China . Email: ben.liu@njnu.edu.cn ; Email: baojianchun@njnu.edu.cn; b Department of Chemistry , Institute of Materials Sciences , University of Connecticut , Storrs , Connecticut 06269 , USA . Email: jiehe@uconn.edu

## Abstract

We report a facile bottom-up synthetic approach to preparing ultrathin two-dimensional (2D) palladium nanosheets (PdNSs) with selectively exposed surface facets. Our synthetic strategy is based on the utilization of the nanoconfined lamellar mesophases of amphiphilic functional surfactants to template the growth of PdNSs in aqueous solution. Preferential adsorption of functional groups (*e.g.*, COOH, pyridyl and quaternary ammonium) and halide counter ions (*e.g.*, Br^–^ and Cl^–^) in the long-chain surfactants onto different Pd planes results in the epitaxial growth of {100}, {110} and {111}-exposed surface facets of ultrathin PdNSs. Our synthetic approach is a general, powerful and scalable method to precisely control the surface facets of ultrathin 2D PdNSs, thus providing an opportunity to evaluate facet-dependent catalytic performance of Pd nanocrystals. Ultrathin PdNSs have been examined as the electrocatalysts for hydrogen evolution reactions (HERs). We show that {100}-exposed PdNSs display superior catalytic activity and stability for HERs, compared to that of {110} and {111}-exposed ones as well as their bulk counterparts. Conceivably, our findings will offer a general guideline in rational design of surfactant templates for other 2D metal nanosheets.

## Abstract 2

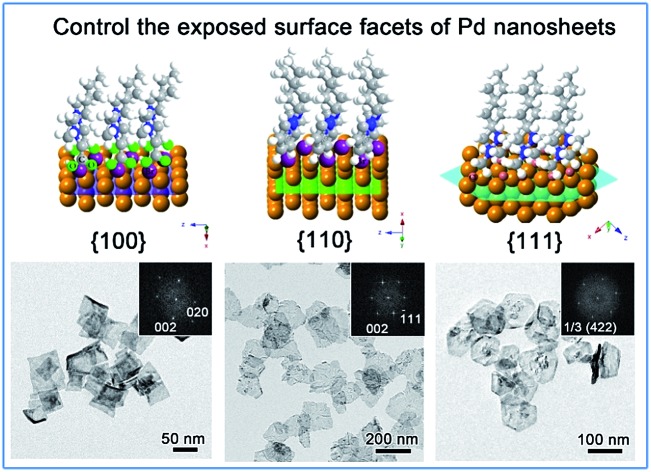
Ultrathin two-dimensional palladium nanosheets with selectively exposed surface facets were controllably synthesized by designed functional surfactants.

## 

Ultrathin two-dimensional (2D) noble metal nanomaterials with atomic thickness have received considerable interest due to their unique electronic, catalytic, electrochemical, and optical properties, compared to their bulk nanoscale counterparts.^1^–^5^ Those 2D nanosheets with a large portion of exposed surface atoms show superior catalytic activity for a number of chemical transformations.^6^,^7^ The surfactant-directed synthetic approach has been utilized to engineer the nanostructures of ultrathin 2D noble metal nanosheets in the presence of specific adsorbates.^8^–^11^ One example is the synthesis of Pd nanosheets with {111}-exposed crystal facet (denoted as PdNSs{111} hereafter) using cetyltrimethylammonium bromide and poly(vinylpyrrolidone) as templates and capping agents, and CO as reductant and facet-selective adsorbate.^12^–^15^ Preferentially strong adsorption of CO onto Pd{111} plane inhibited the growth of Pd along {111} plane, and facilitated the epitaxial growth on uncovered {110} and {100} planes. This resulted in the formation of ultrathin PdNSs{111} with the hexagonal shape.^16^,^17^ Other adsorbates, *e.g.*, halide ions and amines, have also been utilized to assist the control of exposed facets of Pd nanoparticles.^18^–^20^ However, it is largely unsuccessful when extending the methodology to expose other low-index surface facets of ultrathin 2D PdNSs (*e.g.*, {110} or {100}), although there are some examples on controlling the exposed facets in Pd nanocubes, polyhedrons and nanorods.^21^–^24^ This may be originated from the complexity in tuning the interactions between the surfactants/adsorbates and specific Pd planes while simultaneously maintaining confined 2D nanostructures during the synthesis.^19^,^25^ Therefore, a simple and general synthetic methodology to precisely control the specific exposed surface facets of ultrathin 2D PdNSs is exceedingly desired.^18^,^26^

We herein demonstrate a facile yet powerful bottom-up synthesis of ultrathin PdNSs with efficiently controlled surface facets through a template-assisted solution-phase growth. In comparison to the conventional surfactant templates, our design features the precious control of binding between metal precursors and surfactants by tuning the functionality on the head groups of the long-chain amphiphilic surfactants (see Fig. S1 and Table S1† for chemical structures of the surfactants). When varying the hydrophilic head groups, preferential adsorption on different crystal planes can direct the epitaxial growth along plane directions to control the exposed surface facets of PdNSs. Three functional groups, including carboxyl (COOH), pyridyl (Py) and quaternary ammonium (QA), are designed on surfactants having different lengths of alkyl tails to tune the surface crystal facets of 2D PdNSs. The functional surfactants used as the templates include three structural features: (i) the long-chain alkyl hydrophobic tails (*i.e.* C_22_) that direct the self-assembly into lamellar mesophases, (ii) the hydrophilic functional heads which stabilize the lamellar mesophases, bind/confine metal precursors with specific planes, and drive the epitaxial growth along plane directions into ultrathin 2D nanosheets, and (iii) the halide counter ions that assist the control of the exposed surface facets of 2D nanosheets (see Fig. 1a, b, S2 and S3†). Our synthetic method does not require any other additional adsorbates. As shown in Fig. 1b, all three types of the low-index exposed facets of {100}, {110} and {111} in ultrathin 2D nanostructures have been successfully obtained using a family of the surfactants with various functional groups of COOH, Py and QA, respectively. Facet-dependent catalytic performances of as-resultant PdNSs were finally evaluated as the electrocatalysts for hydrogen evolution reactions (HERs).

**Fig. 1:**
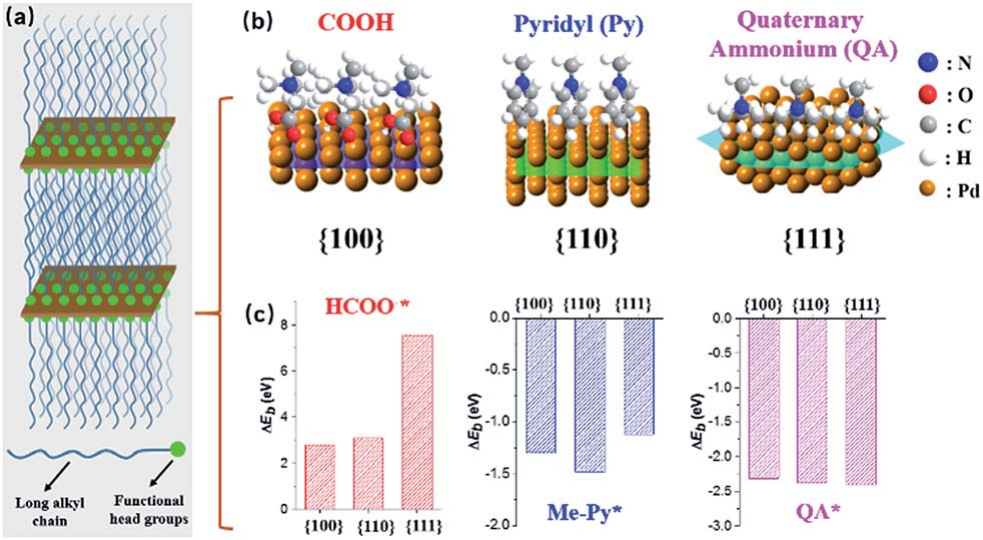
Schematic illustration for surface facet-controlled synthesis of ultrathin PdNSs. (a) Scheme to show the assembled lamellar mesophases between long-chain alkyl surfactants and the growth of Pd nanocrystals. (b) Surface binding behaviours and (c) corresponded Δ*E*_b_ of functional head groups onto different Pd crystal planes based on first-principles calculations.

First-principles calculations were first used to estimate the surface binding behaviors of the functional surfactants on different crystal facets of Pd (Fig. S4†).^27^–^29^ To simplify the simulations, we used COOH, Py and QA with methyl tails to examine the binding energy (Δ*E*_b_) between functional head groups of the surfactants and different Pd crystal planes (see details in ESI†). A more negative Δ*E*_b_ is indicative of thermodynamically favourable binding. Among three functional groups, a more negative Δ*E*_b_ is observed for COOH-containing surfactants preferentially bound to Pd{100}plane, compared to {110} and {111} planes (Fig. 1c). Similarly, Py-containing surfactants favor the binding to Pd{110} plane. Nearly no differences for QA-containing surfactants on three planes were seen. Based on above rationales from simulation, those three groups have been examined to modify the bulky surfactants to tune the surface facets of {100}, (110) and {111} in PdNSs.

PdNSs{100}, which has not been experimentally synthesized, were grown using COOH-containing surfactants of C_*n*_H_2*n*+1_N^+^(CH_3_)_2_CH_2_COOH (Br^–^) (C_*n*_N–COOH (Br^–^), *n* = 14–22) as templates. COOH group of surfactants thermodynamically favors the adsorption onto Pd{100} planes.^30^–^32^ The synthetic details of the surfactants and ultrathin PdNSs{100} are given in ESI (see Fig. S5† for time-dependent experimental). In a typical synthesis, the Pd precursor (H_2_PdBr_4_, obtained by the rapid ligand exchange between PdCl_4_^2–^ and Br^–^)^33^ was mixed with C_22_N–COOH (Br^–^) in aqueous solution, to direct the self-assembly into lamellar mesophases at 35 °C (see Fig. S3† for small-angle X-ray diffraction (XRD) and small-angle X-ray scattering (SAXS)). Then, the fresh-prepared ascorbic acid was injected into the above solution to start reduction of PdBr_4_^2–^ into metallic Pd *in situ*. The color of reaction mixture changed from light-brown, to blue and eventually to dark-blue in 3 h, indicative of the formation of Pd nanocrystals. The solution-phase synthetic route is in one step, and it can be easily scale-up to 1 L (Fig. S3†).

The nanostructures of as-resultant PdNSs{100} are revealed by electron microscopy. As shown in Fig. 2, the representative low-magnification scanning electron microscopy (SEM) and transmission electron microscopy (TEM) images clearly show the ultrathin nanosheets of Pd with a high purity and uniformity (see Fig. S6 and S7† for more low-magnification SEM and TEM images). PdNSs{100} are in nearly square shapes with an average edge length of ∼80 nm (Fig. 2i), although slightly curly edges with higher contrast can be seen. The square morphology of ultrathin 2D PdNSs is confirmed by high-angle annular dark-field scanning TEM (HAADF-STEM) and corresponded elemental mapping (Fig. 2c). High-resolution TEM (HRTEM) of PdNSs shows the square lattice fringes with an inter-planar spacing of 1.96 Å (Fig. 2d), which can be assigned to the {200} plane of face-centered cubic (fcc) Pd (see wide-angle XRD in Fig. 2h). Fourier transform (FT) pattern further confirms the growth in [100] plane direction. We examined the lattice fringes of >20 different nanosheets (Fig. S9†) and all nanosheets show identical crystal structures, suggesting the same exposed surface facet of PdNSs. HRTEM images of different domains in an individual PdNS exhibits uniform spacings and orientations of the lattice fringes (Fig. S10†), indicating the single-crystalline feature of PdNSs. Selected-area electron diffraction (SAED) pattern in Fig. 2e displays a series of spots of single-crystalline structure, corresponding to the [100] zone diffraction of the fcc Pd. The thickness of PdNSs is measured to be *ca.* 2.5 nm with ∼12 atomic layers, when taking TEM images perpendicular to the nanosheet (Fig. 2f). The fringes with a lattice spacing of 1.99 Å can be indexed to the Pd {002}, further indicating that the flat nanosheet planes have exposed {100} facet. The high-resolution X-ray photoelectron spectroscopy (XPS) of Pd 3d shows two asymmetric peaks at 335.4 and 340.6 eV (Fig. 2h), slightly higher than the values of PdNSs capped by quaternary ammonium groups or pyridyl groups (Fig. S11†) and the reported value of commercial Pd nanoparticles,^34^ possibly because of strong surface dipole induced by carboxyl groups. Those findings suggest the first successful preparation of ultrathin PdNSs with {100}-exposed surface facet using the functional surfactant of C_22_N–COOH (Br^–^) simultaneously as the templates and capping agents.

**Fig. 2:**
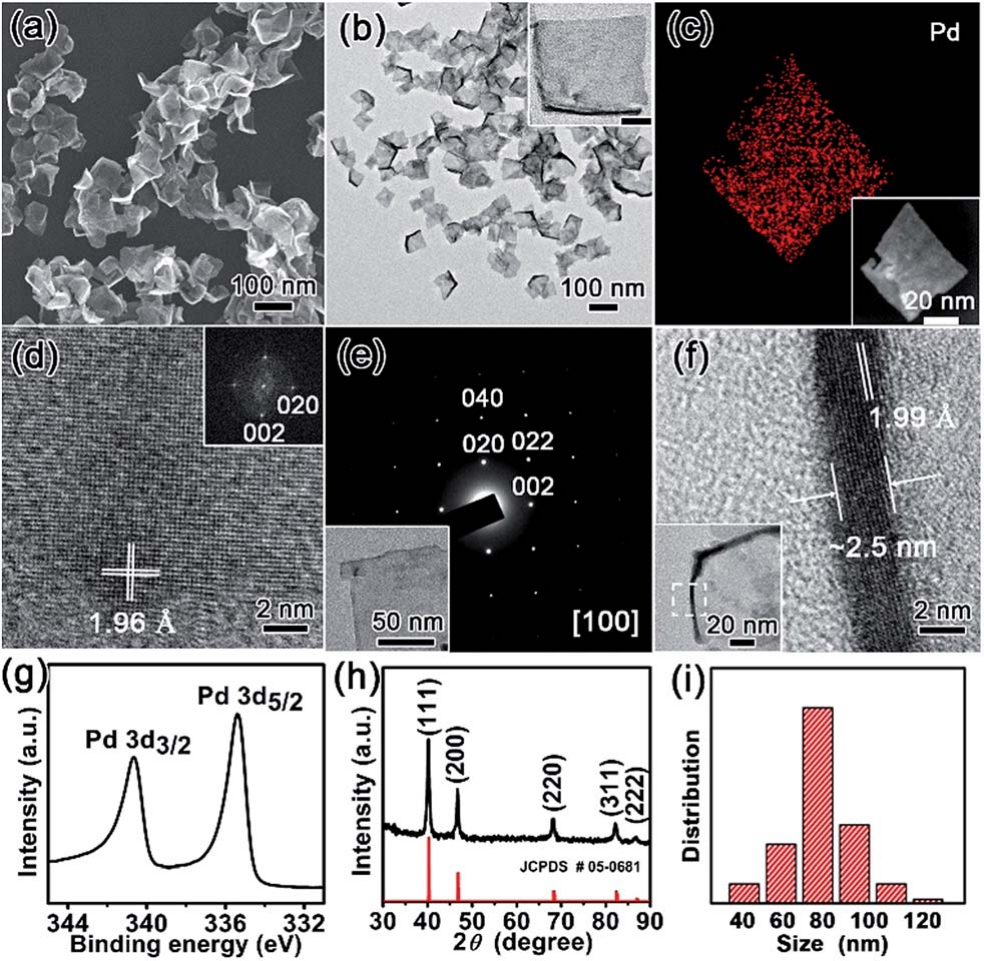
Structural characterizations of ultrathin 2D PdNSs{100} synthesized using C_22_N–COOH (Br^–^). (a) Low-magnification SEM and (b) TEM images of PdNSs{100}. Inserted in (b) is TEM image of an individual PdNS and the scale bar is 20 nm. (c) STEM image and elemental mapping of PdNSs{100}. (d) HRTEM image and corresponded FT pattern of PdNSs{100}. (e) SAED pattern of an individual PdNSs{100} (shown in the inset). (f) HRTEM image taken from the curly edge of a PdNS (the insert) which stands vertically on the TEM grid. (g) Pd 3d XPS spectrum, and (h) wide-angle XRD pattern of PdNSs{100}. (i) The average size and its distribution of PdNSs{100}.

To synthesize PdNSs with {110} and {111}-exposed surface facets, a set of functional surfactants were further examined by varying binding interactions of hydrophilic head groups with Pd crystal planes. Surfactants having functional Py groups that favor to interact the Pd{110} crystal planes,^35^–^37^ were designed (C_22_–Py (Br^–^)). The as-designed functional surfactant of C_22_–Py (Br^–^) thus directed the growth of ultrathin PdNSs{110}. On the other hand, the surfactants with only QA groups and Cl^–^ as counter ion (C_22_–QA (Cl^–^)) were designed to decrease the chemisorption interactions with the Pd{100} or {110} planes, and to expose the Pd{111} planes with low surface energy.^38^ In addition, CO with strong preferential chemisorption onto the Pd{111} plane was also investigated as a control. The formation of ultrathin PdNSs with {111}-exposed facet regardless of the functional groups and halide ions in the surfactants (see Fig. S12 and S13†).

Low-magnification TEM images show that PdNSs{110} and PdNSs{111} are ultrathin with similar sheet nanostructures as PdNSs{100} (Fig. 3a and d). PdNSs{110} are irregular polygons with an average size of ∼150 nm, while PdNSs{111} are nearly hexagonal of ∼100 nm. HRTEM was used to characterize the crystallinity and exposed surface facets of PdNSs. As shown in Fig. 3b, for PdNSs{110}, there are two lattice fringes with the inter-planar spacings of 2.28 and 1.94 Å, ascribed to (111) and (200) planes of fcc Pd. This indicates the [110] growth direction of PdNSs. Meanwhile, the FT pattern (Fig. 3c) also confirms the [110] axes perpendicular to the nanosheet planes. More HRTEM observations of the PdNSs further demonstrated that all nanosheets were enclosed by the {110} plane (see Fig. S14†). For PdNSs{111}, the HRTEM image in Fig. 3e shows an interplanar lattice fringe of 2.42 Å, well-matched to the 1/3 (422) reflection of fcc Pd (Fig. 3e). The result indicates that the PdNSs have exposed {111} facets, similarly in the FT pattern (Fig. 3f).

**Fig. 3:**
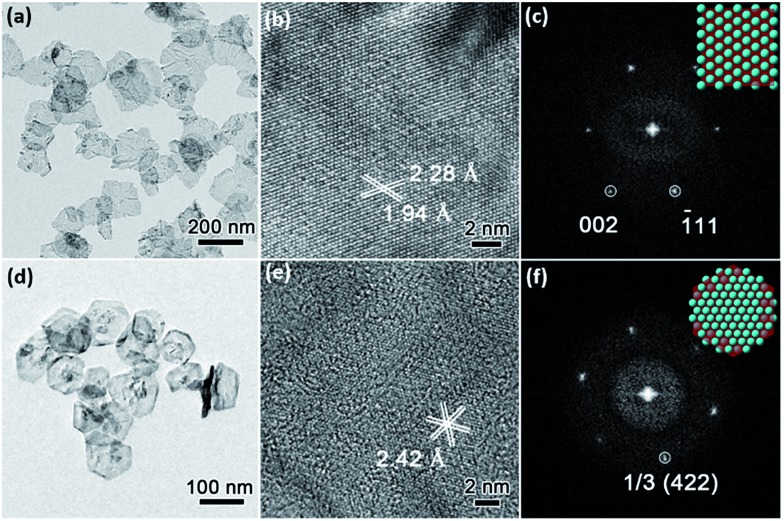
Structural characterizations of (a–c) ultrathin PdNSs{110} and (d–f) PdNSs{111}. (a) TEM, (b) HRTEM images, and (c) corresponded FT pattern of PdNSs{110} synthesized using C_22_–Py (Br^–^). (d) TEM, (e) HRTEM images, and (f) corresponded FT pattern of PdNSs{111} synthesized using C_22_–QA (Cl^–^). The inset in (c) and (f) are schemes indicating the exposed surface facets.

We extended our investigations to understand whether the alkyl lengths and reaction temperatures affect the nanostructures of as-resultant PdNSs. As shown in Fig. 4a, the longer alkyl length favors to stabilize lamellar structures assembled from functional surfactants, promoting the formation of ultrathin 2D nanosheets (see Fig. S15–S17† for TEM images). Similarly, the more ordered nanosheet structures were obtained at the lower reaction temperature (Fig. S18†). This may be because the shorter alkyl chains and the higher reaction temperature will destabilize the initial lamellar mesophases thermodynamically, thus disrupting the epitaxial growth of ultrathin PdNSs along plane direction.^39^,^40^

**Fig. 4:**
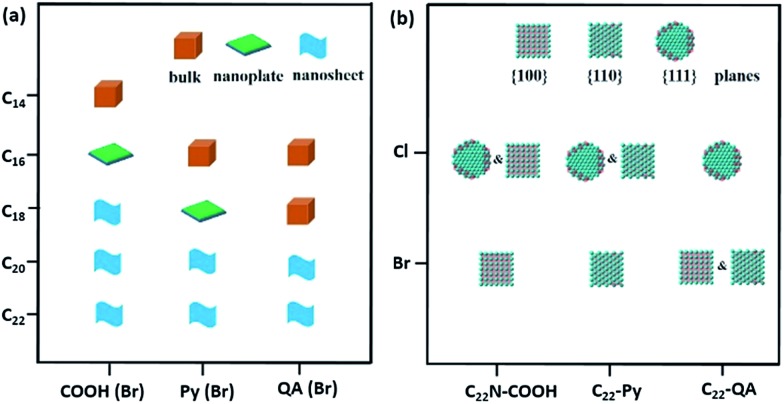
Growth diagrams of Pd nanocrystals synthesized using different surfactants. (a) Structural “phase-like” diagram of Pd nanocrystals obtained from different alkyl lengths and functional groups. (b) Exposed surface facet “phase-like” diagram of PdNSs collected from different functional groups and halide counter ions.

In addition, the effect of functional head groups and halide counter ions of the surfactants on surface facets of the PdNSs were systematically investigated. To this end, surfactants with long alkyl chain of C_22_ were used to form stable lamellar mesophases; while the head groups and halide counter ions were varied (Fig. 4b). As reported previously, halide ion of Br^–^ preferentially chemisorbed onto Pd{100} and Pd{110} planes, while no favorable binding for Cl^–^.^16^,^32^,^41^ We found that C_22_–QA (Br^–^) led to the formation of PdNSs with the mixed surface facets of {100} and {110} planes with a ratio of 39 : 61, confirming the preferential interaction of Br^–^ ions with Pd{100} or {110} planes (Fig. S19†). Those findings suggest that the weak adsorption of both quaternary ammonium groups and Cl^–^ ions on Pd crystal planes is the key to the formation of PdNSs with the lower crystal facets of Pd{111}. On the contrary, when Cl^–^ was used instead of Br^–^ in the surfactants of C_22_N–COOH (Cl^–^) and C_22_–Py (Cl^–^), PdNSs{111} mixed with PdNSs{100} ({111} : {100} = 46 : 54) and PdNSs{110} ({111} : {110} = 32 : 68) were simultaneously observed (Fig. S20†). Given slight difference of Δ*E*_b_ in COOH and Py-containing surfactants (Fig. 1c), the results further suggested that the halide ion of Br^–^ has a synergistic effect on controlling exposed facets of the resultant PdNSs.

Electrocatalytic HERs were further used as a model reaction to evaluate the surface facet-dependent catalytic performances using PdNSs as the electrocatalysts. The commercial Pt (cPt) and Pd black (PdB) (see TEM images in Fig. S21†) were also tested as controls. Fig. 5a shows HER linear sweep voltammetry (LSV) curves under acidic condition (0.5 M H_2_SO_4_) at a scan rate of 5 mV s^–1^. PdNSs with different exposed surface facets exhibited completely different HER activities, following an order of PdNSs{100} > PdNSs{110} > PdNSs{111}. The lowest overpotential of 67 mV was seen for PdNSs{100} at a current density of 10 mV cm^–2^, which is 91 and 160 mV lower than that of PdNSs{110} and PdNSs{111}, respectively (Fig. 5b). Besides, PdNSs{100} exhibited a comparable HER activity to the state-of-the-art catalyst of cPt (the overpotential of PdNSs{100} is only 49 mV higher than that of cPt at 10 mV cm^–2^). The similar trends of HER activities were also observed at current densities at 50 and 100 mV cm^–2^ (Fig. 5b). The better electrocatalytic performance of PdNSs{100} may be originated from the optimal balance between adsorption and desorption of H on the Pd{100} planes during HER.^42^,^43^ By contrast, commercial PdB exhibited the lowest HER activity due to their large sizes.

**Fig. 5:**
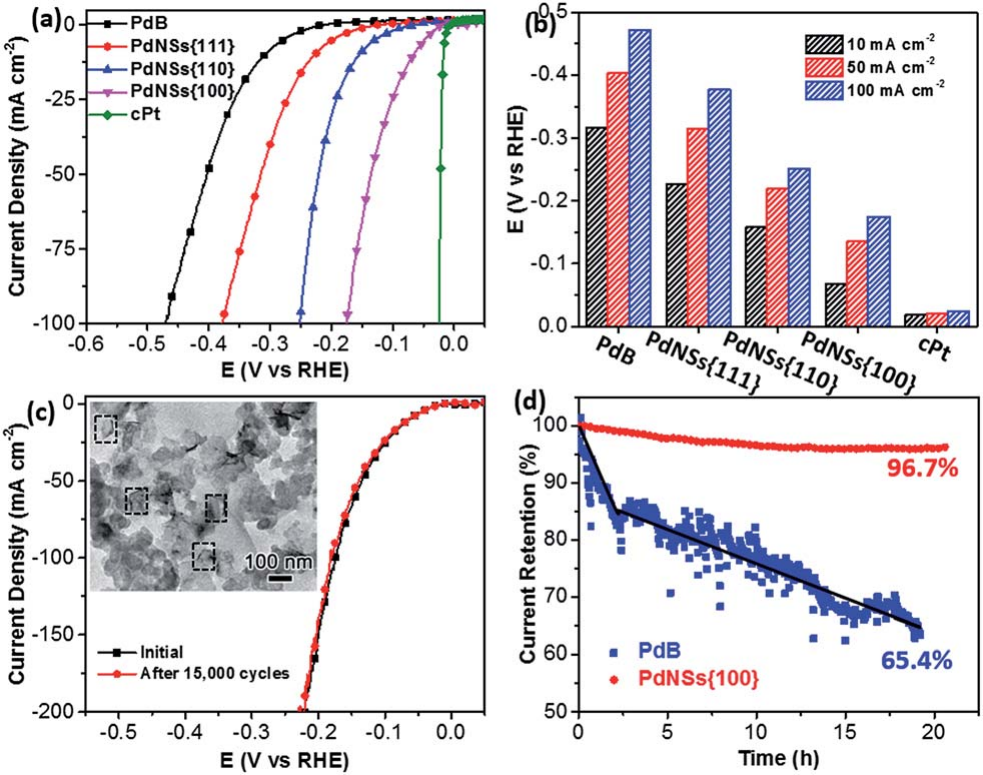
Electrocatalytic HER performances of different PdNSs in 0.5 M H_2_SO_4_. (a) LSV curves of PdNSs{100}, PdNSs{110}, PdNSs{111}, PdB and cPt at a scan rate of 5 mV s^–1^. (b) Electrocatalytic HER activities of PdNSs{100}, PdNSs{110}, PdNSs{111}, PdB and cPt at current density of 10, 50 and 100 mA cm^–2^, respectively. (c) LSV curves of PdNSs{100} before and after repeating CV scans of 15 000 cycles at 50 mV s^–1^. Inserted in (c) is the TEM image of PdNSs{100} after test for 15 000 cycles. (d) Time-dependent *i*–*t* curves of PdNSs{100} and commercial PdB under a constant overpotential of 42 mV at 50 mA cm^–2^.

The electrocatalytic stability of ultrathin 2D PdNSs{100} was further examined. As shown in Fig. 5c, negligible shifts of LSV curves were observed for PdNSs{100} after continuous potential sweeps for 15 000 cycles. The result indicated the superior stability of ultrathin PdNSs. No significant change on the nanosheets shape of the PdNSs{100} was also observed after test for 15 000 cycles (see the TEM image as the inset in Fig. 5c), further confirming the superior stability. The current–time (*i*–*t*) chronoamperometric response was also recorded by a continuous potential of 42 mV at the current density of 50 mA cm^–2^ (Fig. 5d). A respectable current retention of 96.7% was maintained for 21 h, further indicating the superior electrocatalytic durability of PdNSs{100} in an acidic solution. In contrast, only 65.4% of current retention was maintained (∼20 h) for commercialized PdB nanoparticles. The improved electrocatalytic stability of ultrathin PdNSs can be ascribed to the anisotropic ultrathin 2D nanostructures which retarded the dissolution and ripening processes of PdNSs and strengthened the physical interaction between catalysts and the carbon support/electrode.^1^,^44^

In summary, we reported a novel but facile surfactant-assisted synthetic approach for epitaxial growth of ultrathin 2D nanosheets with well-controlled exposed surface facets, for the first time. The key to the synthesis of ultrathin PdNSs with specific exposed facets is the utilization of surfactants having functional hydrophilic head groups as the templates and capping agents. The change in hydrophobic carbon chain lengths, functional head groups and halide counter ions of the surfactants resulted in specifically exposed surface facets of ultrafine metal nanosheets. As a result, all three low-index exposed crystal facets of {100}, {110} and {111} in ultrathin PdNSs were synthesized using the surfactant of C_22_N–COOH (Br^–^), C_22_–Py (Br^–^) and C_22_–QA (Cl^–^), respectively. Lastly, the surface facet-dependent electrocatalytic activity of ultrathin PdNSs was also examined by HERs. We found that the PdNS{100} exhibited much better electrocatalytic activity and stability, compared to the PdNS{110} and PdNS{111}. This work represents the first successful example for facet-controlled synthesis of ultrathin PdNSs. We believe that the concept of 2D-templated strategy deriving from the self-assembly of novel designed functional surfactants could provide new insight in the prospective construction of other kinds of ultrathin noble metals or related inorganic nanomaterials.

## Conflicts of interest

There are no conflicts to declare.

## Supplementary Material

Supplementary information
