# Supplementary material for: Ultrathin palladium nanosheets with selectively controlled surface facets
Source: Chem Sci. 2018 Apr 2;9(19):4451–5. doi: 10.1039/c8sc00605a (PMC5956979; doi:10.1039/c8sc00605a)
Supplement: Supplementary file 1 [file SC-009-C8SC00605A-s001.pdf]

Electronic Supplementary Material (ESI) for Chemical Science.
This journal is © The Royal Society of Chemistry 2018

Ultrathin Palladium Nanosheets with Selectively Controlled Surface Facets

Dongdong Xu,[a] Xiaoli Liu,[a] Hao Lv,[a] Ying Liu,[a] Shulin Zhao,[a] Min Han,[a] Jianchun Bao,[a] Jie He,*[b] and Ben Liu*[a]*

[a]Jiangsu Key Laboratory of New Power Batteries, Jiangsu Collaborative Innovation Center of Biomedical Functional Materials, School of Chemistry and Materials Science, Nanjing Normal University, Nanjing, Jiangsu 210023, China

[b]Department of Chemistry, and Institute of Materials Science, University of Connecticut, Storrs, Connecticut 06269, United States.

Email: ben.liu@njnu.edu.cn; baojianchun@njnu.edu.cn; jie.he@uconn.edu

Table of Contents

1. Materials....................2
2. Synthesis of the functional surfactants....................2
3. Synthesis of ultrathin PdNSs....................3
4. First-principles calculations....................3
5. Electrochemical hydrogen evolution reaction (HER)....................4
6. Characterizations....................4

Figure Contents

Supplementary Fig. S1. The molecular structures of the designed functional surfactants.

Supplementary Fig. S2-3. Schematic illustration for the synthesis of ultrathin PdNSs by C_{22}N-COOH (Br^-).

Supplementary Fig. S4. Simulation results of the functional surfactants onto different Pd crystal facets.

Supplementary Fig. S5. Formation mechanism of PdNSs{100}.

Supplementary Fig. S6-11. Structural characterizations of PdNSs{100}.

Supplementary Fig. S12-13. CO-assisted synthesis of ultrathin PdNSs{111}.

Supplementary Fig. S14-20. Structural characterizations of Pd nanostructures synthesized by different surfactants.

Supplementary Fig. S21. Structural characterizations of commercial PdB and cPt nanoparticles.

Table Content

Supplementary Table S1. Summarization of all the sample information and corresponding synthetic conditions.

1. Materials

Palladium (II) chloride (99.9 wt%), L-ascorbic acid (AA) (99%), commercial palladium black and platinum were purchased from Alfa Aesar. Hydrochloric acid, isopropanol, ethanol, acetonitrile, pyridine, and diethyl ether were obtained from Sinopharm Chemical Reagent Co. Ltd. (Shanghai). 1-Bromodocosane (98%), 1-bromoeicosane (95%), 1-bromooctadecane (97%), 1-bromohexadecane (96%), 1-bromotetradecane (97%), and 1-chlorodocosane (98%) were purchased from TCI Corporation. *N,N*-dimethyldocosylamine, *N,N*-dimethyleicosylamine, *N,N*-dimethyloctadecylamine, *N,N*-dimethylhexadecylamine, *N,N*-dimethyltetradecylamine were purchased from Heowns Opde Technologies (Tianjin). Trimethylamine (~25% in methanol), chloroacetic acid (99%), bromoacetic acid (99%) were obtained from Adamas-beta. H_2PdCl_4 solution (10 mM) was prepared by dissolving 0.36 g of palladium (II) chloride in 20 mL of HCl solution (0.2 M) and further diluting to 200 mL with deionized water. All the reagents were of analytical reagent grade and used without further purification.

2. Synthesis of the functional surfactants

2.1. Synthesis of C_nN-COOH (Br^-) and C_{22}N-COOH (Cl^-). In a typical synthesis of C_{22}N-COOH (Br^-), 3.76 g of *N,N*-dimethyldocosylamine (10 mM) and 1.24 g of bromoacetic acid (11 mM) were mixed in 50 ml of isopropanol and then refluxed under 95 °C for 24 h. After the removal of solvent by reduced pressure distillation, the crude product was washed with diethyl ether several times and dried in a vacuum oven overnight. The final white product of C_{22}N-COOH (Br^-) was accordingly obtained before use. The other surfactants with different alkyl length were synthesized by substituting *N,N*-dimethyldocosylamine with *N,N*-dimethyleicosylamine, *N,N*-dimethyloctadecylamine, *N,N*-dimethylhexadecylamine, *N,N*-dimethyltetradecylamine, respectively, *via* the similar procedures. C_{22}N-COOH (Cl^-) was synthesized by substituting bromoacetic acid with chloroacetic acid and following the above-mentioned procedures. The products were verified by ^{1}H NMR. Taking C_{22}N-COOH (Br^-) for an example (CD_3OD): δ 4.15 (s, 2H), 3.29 (s, 6H), 2.94 (m, 2H), 1.68 (m, 2H), 1.42-1.20 (m, 38H), 0.90 (t, J = 6.6 Hz, 3H).

2.2. Synthesis of C_n-QA (Br^-) and C_{22}-QA (Cl^-). In a typical synthesis of C_{22}-QA (Br^-), 3.9 g of 1-bromodocosane (10 mM) and 3.54 g of trimethylamine (15 mM) were mixed in 150 ml of acetonitrile and further refluxed under 95 °C for 20 hours. After cooling to room temperature, the solvent was removed by reduced pressure distillation. Then, the crude product was washed with diethyl ether three times and dried in a vacuum oven overnight. The cationic surfactants with different alkyl length (C_{20}-QA (Br^-), C_{18}-QA (Br^-), and C_{16}-QA (Br^-)) were obtained following the similar procedures by substituting 1-

bromododocosane with 1-bromoeicosane, 1-bromooctadecane, and 1-bromohexadecane, respectively. C_{22}-QA (Cl^-) was synthesized using 1-chlorodocosane and trimethylamine through the above-mentioned procedures. ^{1}H NMR (CD_3OD) of C_{22}-QA (Br^-): δ 3.33 (d, J = 14.4 Hz, 2H), 3.12 (s, 9H), 1.78 (m, 2H), 1.48-1.16 (m, 38H), 0.90 (t, J = 6.8 Hz, 3H).

2.3. Synthesis of C_n-Py (Br^-) and C_{22}-Py (Cl^-). In a typical synthesis of C_{22}-Py (Br^-), 3.9 g of 1-bromodocosane (10 mM) and 1.2 g of pyridine (15 mM) were mixed in 200 ml of acetonitrile and then refluxed under 95 ^oC for 20 hours. After cooling to room temperature, the solvent was removed by reduced pressure distillation. Then, the crude product was washed with diethyl ether and dried in a vacuum oven overnight. The pyridyl-type surfactants with different alkyl length (C_{20}-Py (Br^-), C_{18}-Py (Br^-), and C_{16}-Py (Br^-)) were also obtained following the similar procedures by substituting 1-bromodocosane with 1-bromoeicosane, 1-bromooctadecane, 1-bromohexadecane, and 1-bromotetradecane, respectively. C_{22}-Py (Cl^-) was synthesized by using 1-chlorodocosane through the above-mentioned procedures. ^{1}H NMR (CD_3OD) of C_{22}-Py (Br^-): δ 9.02 (d, J = 5.7 Hz, 2H), 8.59 (m, 1H), 8.12 (t, J = 7.0 Hz, 2H), 4.64 (m, 2H), 1.39-1.28 (m, 40H), 0.90 (t, J = 6.9 Hz, 3H).

3. Synthesis of ultrathin PdNSs

In a typical synthesis of the PdNSs{100}, 1.6 mL of H_2PdCl_4 aqueous solution (10 mM) was added into a vial containing 5 mL of C_{22}N-COOH (Br^-) aqueous solution (0.05 mM) at room temperature. After homogeneous mixing, 1 mL of fresh AA aqueous solution (0.3 M) was injected into the above solution. The synthesis composition ratio is C_{22}N-COOH (Br^-): H_2PdCl_4: AA = 25: 1.6: 30. Then the vial was placed undisturbedly at 35 ^oC for several hours. After that, the black product was collected by centrifugation and washed several times with absolute ethanol, and then freeze-dried at -60 ºC. The Pd products synthesized by other surfactants were obtained *via* the similar procedures. Besides, CO-assisted synthesis of PdNSs was carried out by bubbling of CO gas into the reaction solution, instead of the addition of AA (take care when using toxic CO).

4. First-principles calculations

First-principles calculations were performed based on the generalized gradient approximation (GGA) with plane-wave basis sets and ultrasoft pseudopotentials, as implemented in the CASTEP code. The tolerance of the energy was set as 1×10^{-3} eV/cell. The exchange-correlation energy and potential were described self-consistently using the Perdew, Burke, and Ernzerhof (PBE) functional. Brillouin zone integration was performed with variable number of *k*-points generated by Monkhorst-Pack algorithm, depending on the cell size and shape. In order to simplify the simulations, we used $HCOO^{-}$*, Py-N^+-Me* and Me_4-N^{+}* instead of C_{22}N-COOH, C_{22}-Py and C_{22}-QA, respectively. For the supercells of Pd{100} and Pd{111}, the default value of *k*-point set is 3×3×1, while for the Pd{110} supercell, the default value of *k*-point set is 2×3×1. And the

interaction affinity between the functional head groups (FGs) of the surfactant and Pd planes were elucidated using the binding energy (ΔE_b), i.e., the difference between the total energy of the binding system (E_T(FG/Pd{hkl})) and the sum of energy for the individual Pd plane (E_T(Pd(hkl))) and functional groups (E_T(FG)) as follows:

$$\Delta E_b = E_T(\text{FG/Pd}\{\text{hkl}\}) - E_T(\text{Pd}\{\text{hkl}\}) - E_T(\text{FG}) \quad (1)$$

The configuration of FGs under stable binding state is governed by the ΔE_b of FG with Pd(hkl). A more negative ΔE_b corresponds to the favorite facet for coupling with the specific FGs.

5. Electrochemical hydrogen evolution reaction (HER)

The electrocatalytic tests were performed on the CHI 660E electrochemical analyzer at room temperature. A three-electrodes system was used for all electrochemical tests, which consisted of a carbon rod as the counter electrode, a saturated calomel electrode as the reference electrode, and glassy carbon electrode (GCE, 0.07065 cm^2) as the working electrode. Typically, an ink of the catalysts was prepared by mixing 1 mg of catalysts, 4 mg of carbon black (Vulcan XC-72), 0.8 mL of ethanol and 0.2 mL of water. After sonicating for 30 min, 50 μL of Nafion solution was added and further sonicated for an additional 30 min. Then, 6 μL of the ink solution (~0.006 mg of catalyst) was dropped on the working electrode and dried at room temperature before test. Linear sweep voltammetry (LSV) was used to evaluate the electrochemical activity of different catalysts with a scan rate of 5 mV s^{-1}. All these results were obtained by IR compensation and all reported potentials in this work are referenced to the reversible hydrogen electrode (RHE).

6. Characterizations

The high-resolution thermal-field emission scanning electron microscope (SEM) images were obtained on a JSM-7600F apparatus at an accelerating voltage of 10 kV. SEM samples were prepared by directly casting the suspension of the samples on silicon wafers. The transmission electron microscope (TEM) observations were performed with a JEOL JEM-2100 microscope operated at 200 kV (Cs 0.5 mm, point resolution 1.9 Å). Images were recorded with a Gatan CCD camera (resolution 4000 x 2700 pixels, pixel size 9 x 9 μm). High-angle annular dark-field scanning STEM was taken on JEOL JEM-2100F microscope which are equipped with STEM and EDS detectors for elemental mapping analysis. TEM and STEM samples were prepared by casting a suspension of the samples on a carbon coated copper grid (300 mesh). X-ray diffraction patterns were recorded on powder samples using a D/max 2500 VL/PC diffractometer (Japan) equipped with graphite-monochromatized Cu Kα radiation in 2θ ranging from 30° to 90°. Related work voltage and current were 40 kV and 100 mA,

respectively. ^1H NMR spectra were recorded on Avance III HD 400 spectrometer (400MHz), and the chemical shifts were reported in ppm relative to the residual deuterated solvent and the internal standard tetramethylsilane. The X-ray photoelectron spectra (XPS) were performed on a scanning X-ray microprobe (Thermo ESCALAB 250Xi) that uses Al Kα radiation. The binding energy of the C 1s peak (284.8 eV) was employed as a standard to calibrate the binding energies of other elements. Small-angle X-ray scattering (SAXS) measurements were performed on a SAXSess mc2 apparatus with Cu Kα radiation (Anton Paar).

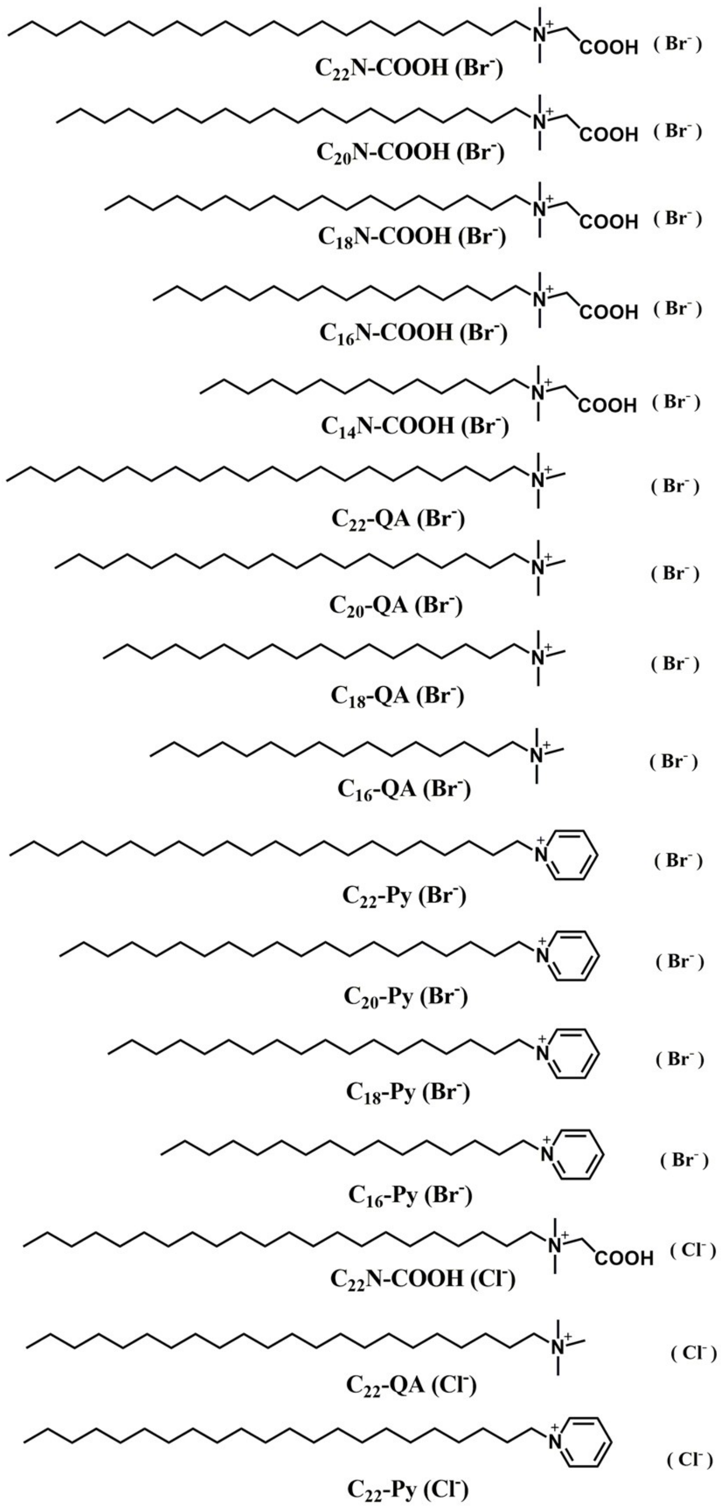

Fig. S1 The molecular structures and the corresponded abbreviations of the designed functional surfactants used in this work.

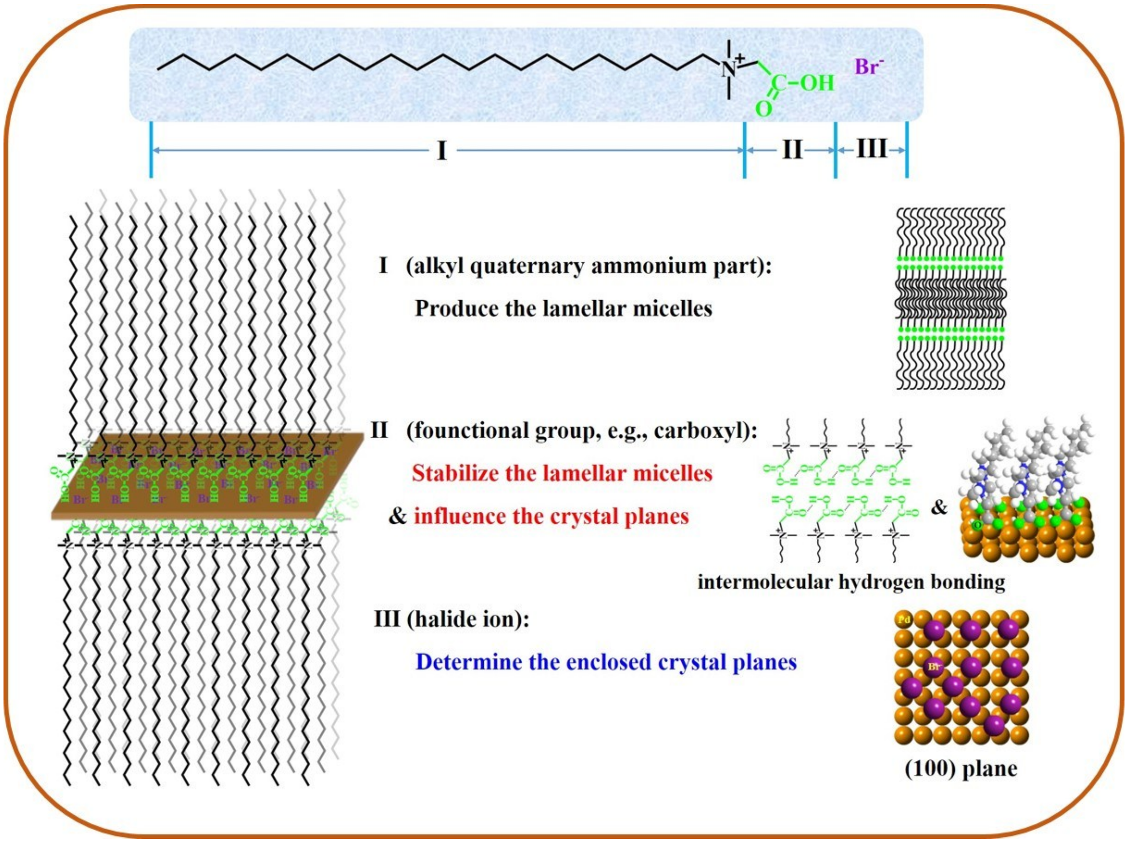

Fig. S2 Schematic illustration for exposed crystal facet-controlled synthesis of ultrathin PdNSs by C_{22}N-COOH (Br^-).

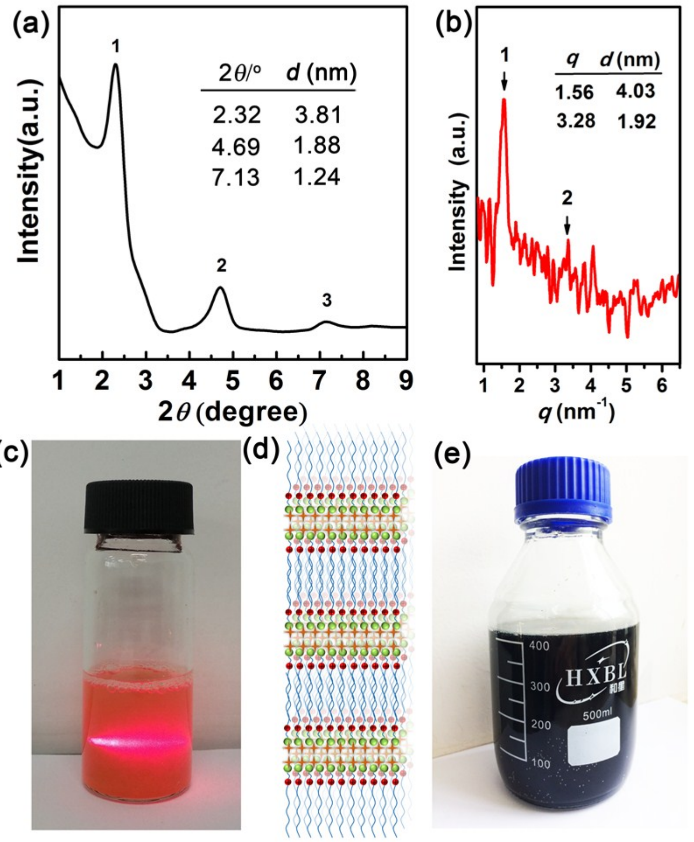

Fig. S3 Structural characterizations of surfactant-$PdCl_4^{2-}$ lamellar assemblies. (a) Low-angle XRD pattern of surfactant-$PdCl_4^{2-}$ hybrids obtained by centrifugation from the synthesis solution consisting of H_2O, $C_{22}N$-COOH (Br^-) and H_2PdCl_4. Three well-defined peaks with the 2θ value of 2.32, 4.69 and 7.13°, with a 1: 2: 3 reciprocal *d*-spacing pattern, were indexed to 100, 200 and 300 diffractions of the typical lamellar mesophase. (b) SAXS spectrum of surfactant-$PdCl_4^{2-}$ solution. Two peaks with the value of q_1: $q_2 \approx$ 1: 2, further confirming lamellar mesophase of the surfactant in aqueous solution. (c) The digital photograph of the surfactant-$PdBr_4^{2-}$ hybrids with Tyndall effect. (d) The schematic illustration of lamellar organic-inorganic surfactant-$PdBr_4^{2-}$ hybrids. (f) The digital photograph of the scale-up synthesis solution.

The lamellar mesophase of surfactant-$PdCl_4^{2-}$ assemblies was confirmed by low-angle XRD in solid and *in situ* SAXS in synthesis solution. Both results show a Bragg peak ratio of 1 : 2 : (3) corresponding to the (100), (200) and (300) (not seen in SAXS) diffraction, indicating the formation of lamellar mesophase when co-assembling surfactant-$PdCl_4^{2-}$ in synthesis solution. The *d*-spacing calculated from SAXS is 4.03 nm, slightly larger than that from low-angle XRD (3.81 nm). This is likely because of the structural shrinkage of the lamellar phase after the removal of water. The similar lamellar mesophases assembled from the surfactant were also observed to synthesize two-dimensional Au nanosheets (*Nat. Commun.*, 2014, **5**, 3313; *J. Am. Chem. Soc.* 2013, **135**, 12544) and lamellar zeolites (*Nature*, 2009, **461**, 246; *Chem. Mater.* 2011, **23**, 5131) *in situ*."

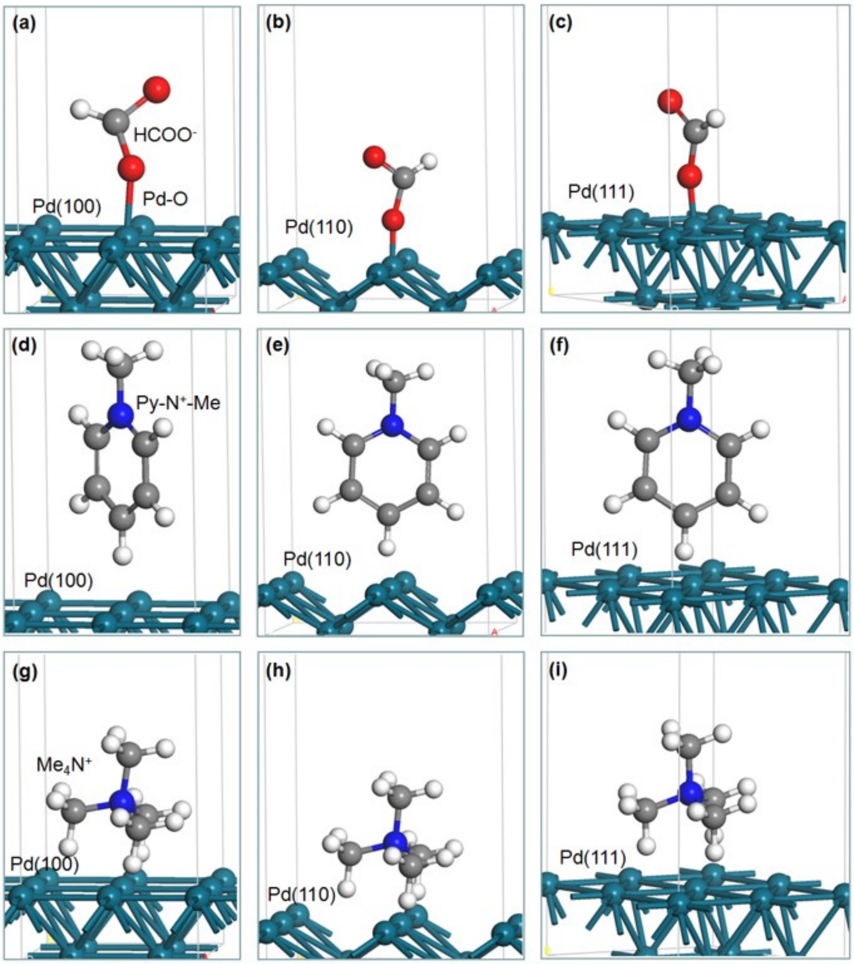

Fig. S4 Simulation results. Binding behaviors of (a-c) HCOO^{-*}, (d-f) Me-Py* and (g-i) QA* onto different Pd crystal planes ({100}, {110} and {111}, respectively).

The configuration of function groups (FGs) under stable binding state is governed by the ΔE_b of FG with Pd(*hkl)*. A more negative ΔE_b corresponds to the favorite facet for coupling with the specific FGs. For instance, the HCOO$^{–}$ onto Pd{100} plane presents the ΔE_b of 2.79 eV (Fig. S3a), which is more negative than the HCOO$^{–}$ onto Pd{110} and Pd{111} (Fig. S3b and c). This indicates that the surfactant with the FG of HCOO$^{–}$ favors to bind onto the Pd{100} plane as the efficient capping agent. The chemisorption of HCOO$^{–}$ onto Pd{100} inhibits the growth of PdNSs along Pd{100} plane, and thus facilitates the formation PdNSs{100} with {100}-exposed facets. However, due to the small difference of binding energy onto Pd{100} and Pd{110} planes, the mixed {100}/{110}-exposed facets were also observed when using the surfactants of C_{22}N-COOH (Cl^{-}), totally same to our experimental results. The results also indicate halide ion of Br^{-} also assists the growth of PdNSs{100} with pure {100}-exposed crystal facet. Similarly, the preferential chemisorption of Py groups onto Pd{110} facets was accordingly confirmed (ΔE_b of -1.48 eV for Pd{110} is lowest). However, due to steric hindrance and very weaker affinity of QA onto Pd facets, it is very difficult to distinguish their favorable exposed facets by simulation.

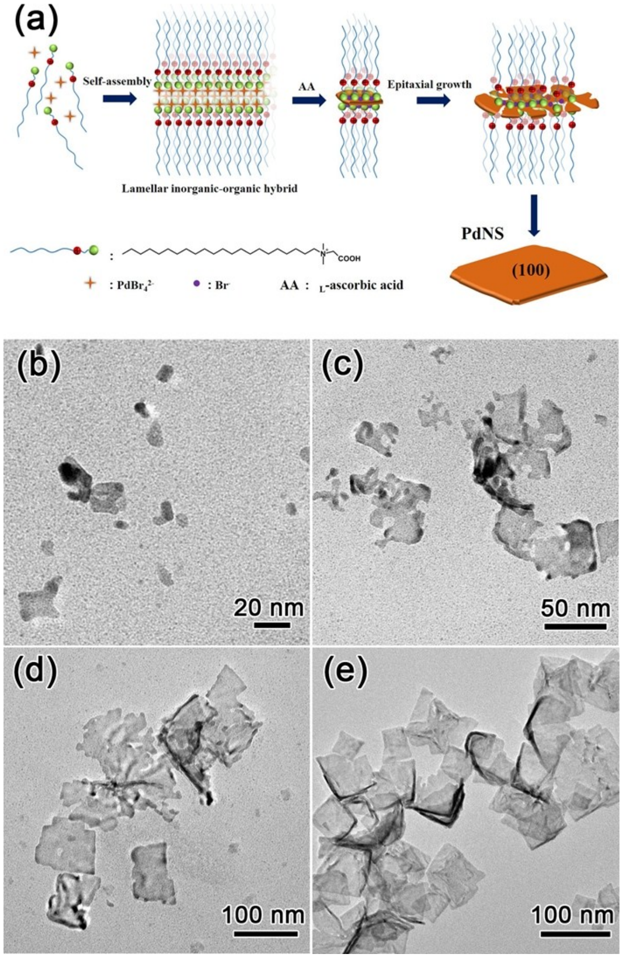

Fig. S5 (a) Schematic illustrating an epitaxial growth mechanism of PdNSs{100} using the surfactant of C_{22}N-COOH (Br^-). (b-e) Representative TEM images of Pd crystals obtained at the different reaction period of (b) 5 min, (c) 30 min, (d) 1 h, and (e) 3 h.

The negatively charged $PdBr_4^{2-}$ would firstly interact with quaternary ammonium group in C_{22}N-COOH (Br^-) through the electrostatic interaction, which further self-assemble into the lamellar organic-inorganic hybrids. The presence of carboxyl groups could greatly stabilize the lamellar micelles due to the intermolecular hydrogen bonding, while the oxygen atoms in carbonyl groups and Br^- would strongly interact and/or adsorb onto Pd {100} plane. Both the nanoconfined effect of lamellar micelles and preferentially chemisorbed planes of Pd determined the construction of ultrathin 2D Pd nanosheets with specific {100}-exposed facets. As indicated by TEM images of PdNSs, small piece of Pd nanosheets (<10 nm) were found at the initial stage (b), which epitaxially grew bigger only along the 2D plane direction (c-e). The irregular edges of the PdNSs intermediates (c, d) gradually grew smooth ones with square morphology, and the size also became bigger with increasing the reaction period. The results indicated the epitaxial growth mechanism of the PdNSs, which also corresponded to the single-crystalline structure and square shape of the as-resulted nanosheets.

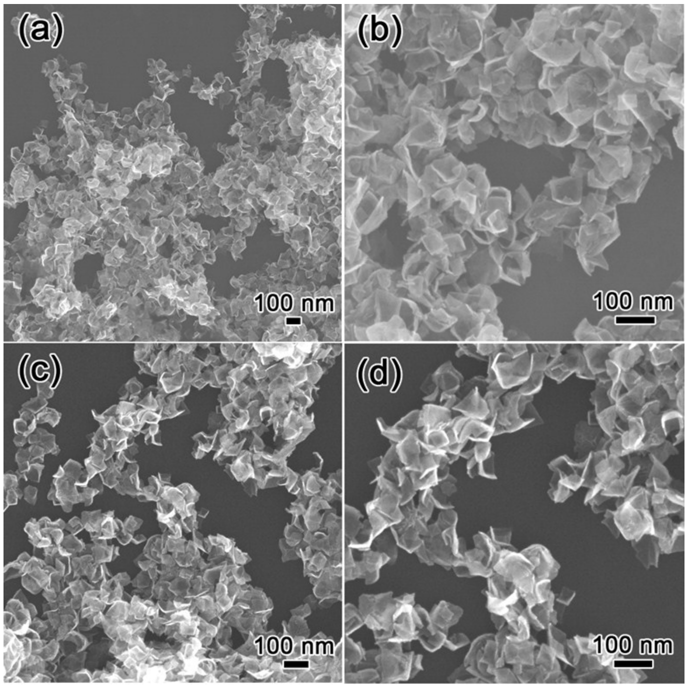

Fig. S6 Supporting SEM images of the PdNSs{100} with different magnifications synthesized using the surfactant of C_{22}N-COOH (Br^-).

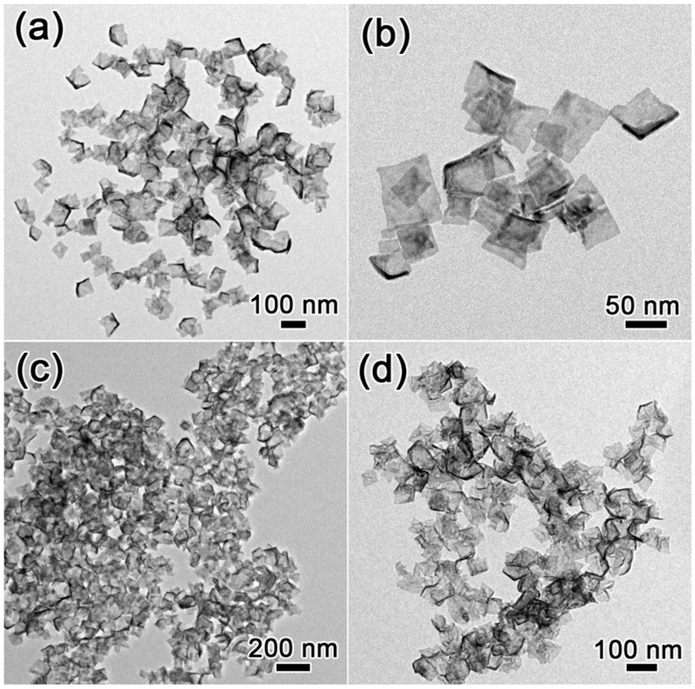

Fig. S7 Supporting TEM images of the PdNSs{100} with different magnifications synthesized using the surfactant of C_{22}N-COOH (Br^-).

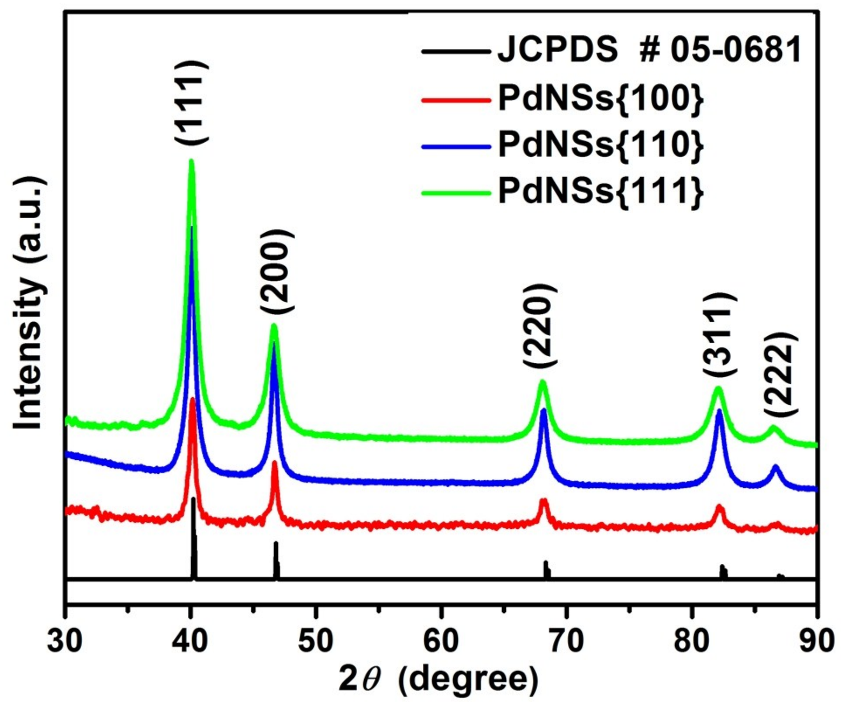

Fig. S8 Wide-angle XRD patterns of PdNSs with different exposed facets. PdNSs{100}, PdNSs{110}, and PdNSs{111} were synthesized using the functional surfactants of C_{22}N-COOH (Br^-), C_{22}-Py (Br^-), and C_{22}-QA (Cl^-), respectively. All of three XRD patterns presented five well-resolved peaks, which were indexed as the (111), (200), (220), (311) and (222) planes of fcc Pd (JCPDS # 05-0681).

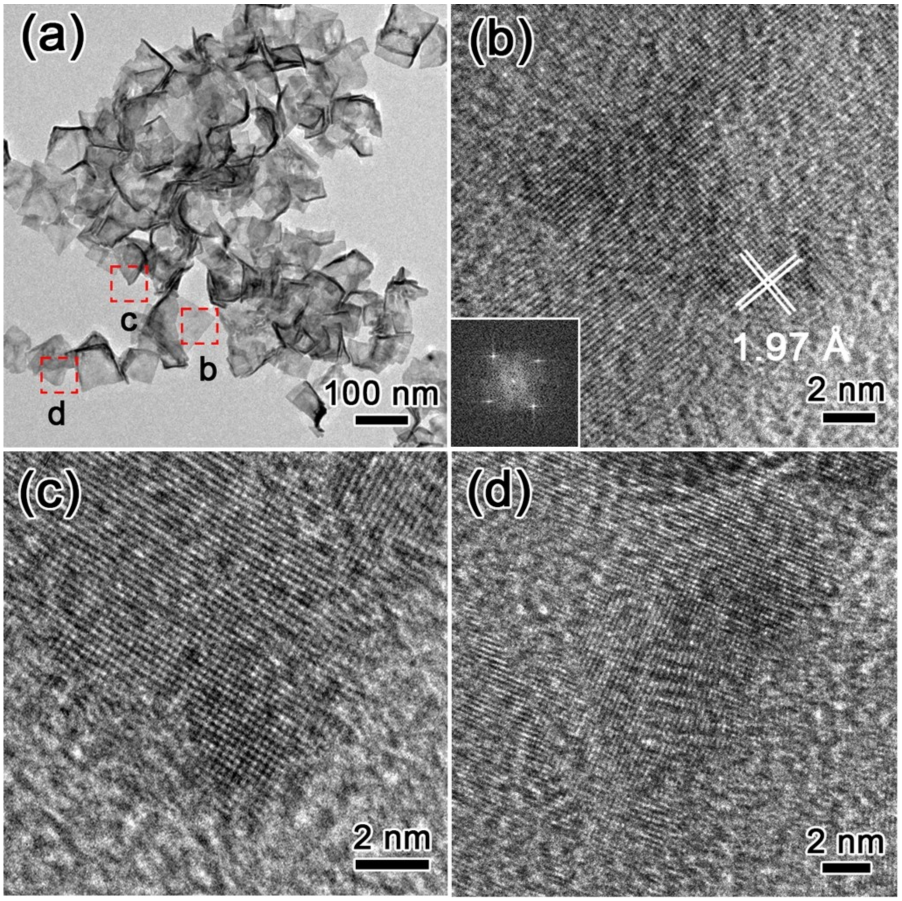

Fig. S9 (a) TEM image of PdNSs{100} and (b-d) typical high-resolution TEM images of different PdNSs labelled in (a), indicating PdNSs composed of the same crystal structures ({100}-exposed facets).

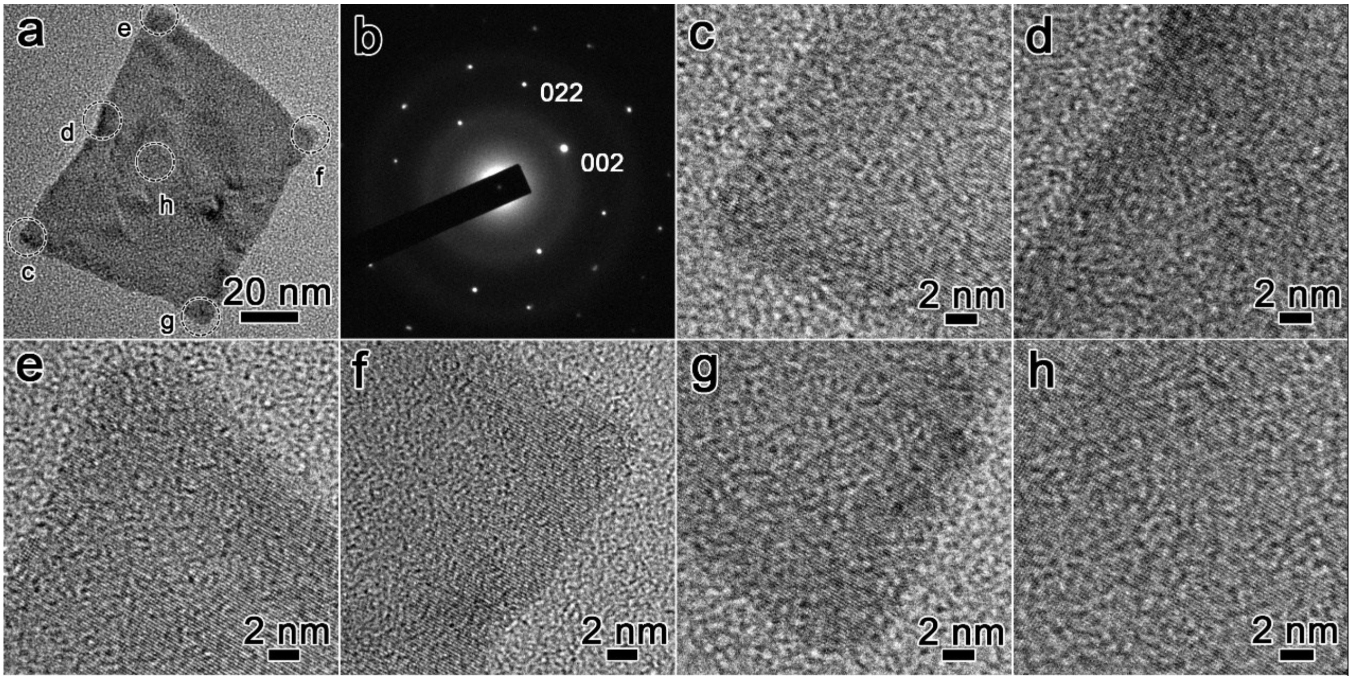

Fig. S10 (a) TEM image and (b) corresponded SAED pattern of an individual PdNSs{100}. (c-h) Typical HRTEM images of different domains in the PdNS labelled in (a). The lattice fringes with the totally same spacing and orientation indicated the single-crystalline structure of as-obtained PdNSs.

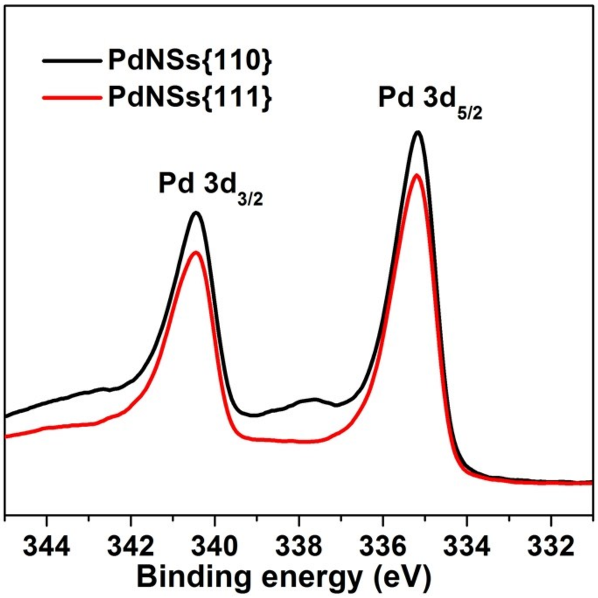

Fig. S11 Pd 3d XPS spectra of ultrathin PdNSs{110} and PdNSs{111}. PdNSs{110} and PdNSs{111} were synthesized using the surfactants of C_{22}-Py (Br^-) and C_{22}-QA (Br^-), respectively.

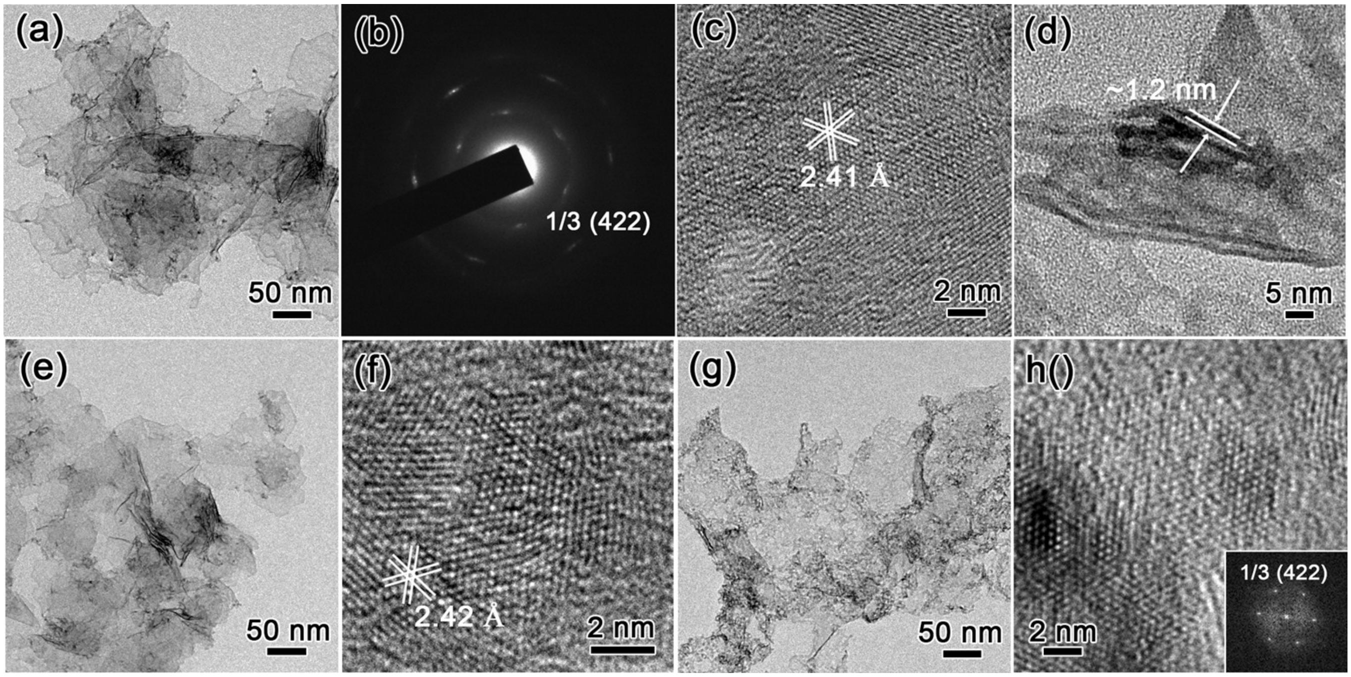

Fig. S12 The structural characterizations of the PdNSs synthesized in the presence of CO gas using the surfactants of (a-d) C_{22}N-COOH (Br^-), (e, f) C_{22}-Py (Br^-) and (g, h) C_{22}-QA (Br^-). (a) TEM and (c) high-resolution TEM image of the PdNSs obtained using C_{22}N-COOH (Br^-) in assistance of CO, and (b) SAED pattern of a single PdNS. The lattice fringes with the *d*-spacing of 2.41 Å could be indexed to the 1/3 (422) reflections of fcc Pd. Both the SAED and the lattice fringes confirmed the formation of Pd nanosheets with {111}-exposed facets. (d) TEM image of the PdNSs which stands vertically on the TEM grid, indicating the thickness of these PdNSs is ~1.2 nm. TEM and high-resolution TEM images of the PdNSs synthesized using (e, f) C_{22}-Py (Br^-) and (g, h) C_{22}-QA (Br^-) in the assistance of CO.

From TEM images, it was observed that, in the assistance of CO, these three kinds of the surfactants (Br^- types) only directed the formation of PdNSs with {111}-exposed facet, because CO molecule strongly adsorbed onto the {111} plane of fcc Pd and thus inhibited the growth of PdNSs along {111} plane direction.

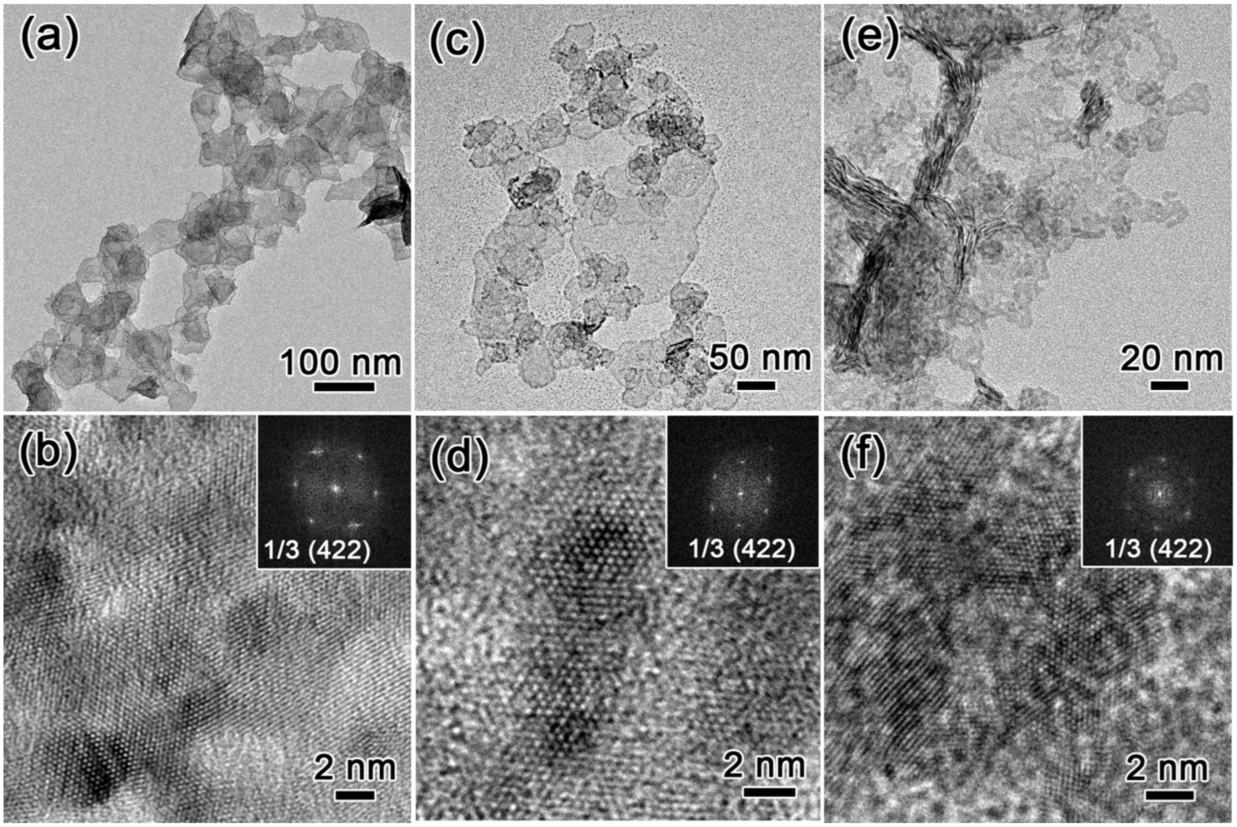

Fig. S13 The structural characterizations of the PdNSs synthesized in the presence of CO gas using the surfactants of (a, b) C_{22}N-COOH (Cl^-), (c, d) C_{22}-Py (Cl^-) and (e, f) C_{22}-QA (Cl^-). (a, c, e) TEM, (b, d, f) high-resolution TEM images and corresponded FT patterns (inserted) of the PdNSs synthesized using the surfactants of (a, b) C_{22}N-COOH (Cl^-), (c, d) C_{22}-Py (Cl^-) and (e, f) C_{22}-QA (Cl^-) in the presence of CO gas. The results indicated that the PdNSs selectively exposed {111} plane facet, regardless of functional groups.

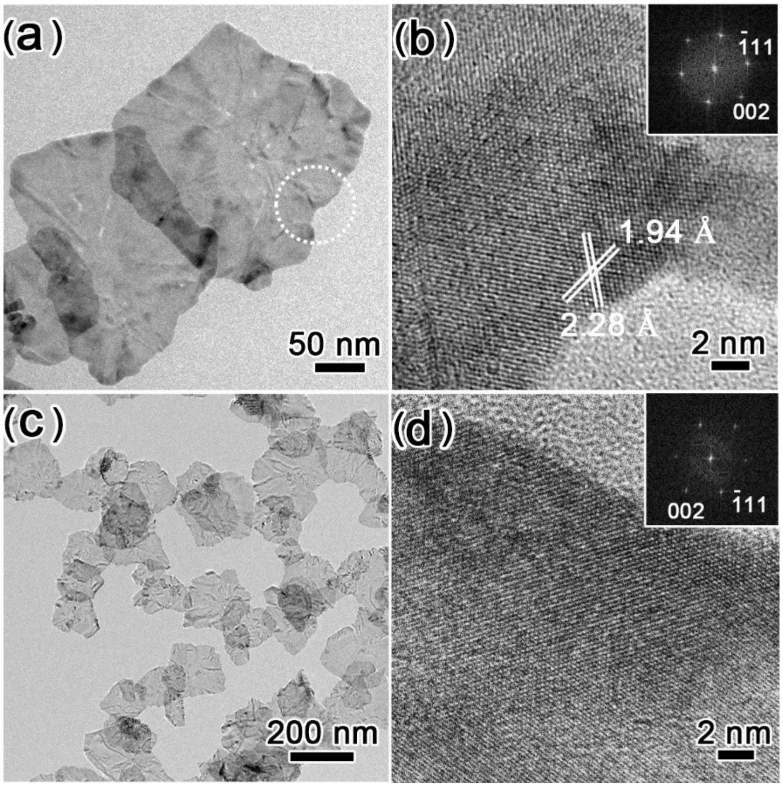

Fig. S14 (a, c) Supporting TEM and (b, d) high-resolution TEM images of PdNSs{110} synthesized using the surfactant of C_{22}-Py (Br^-), indicating the formation of PdNSs with {110}-exposed facet.

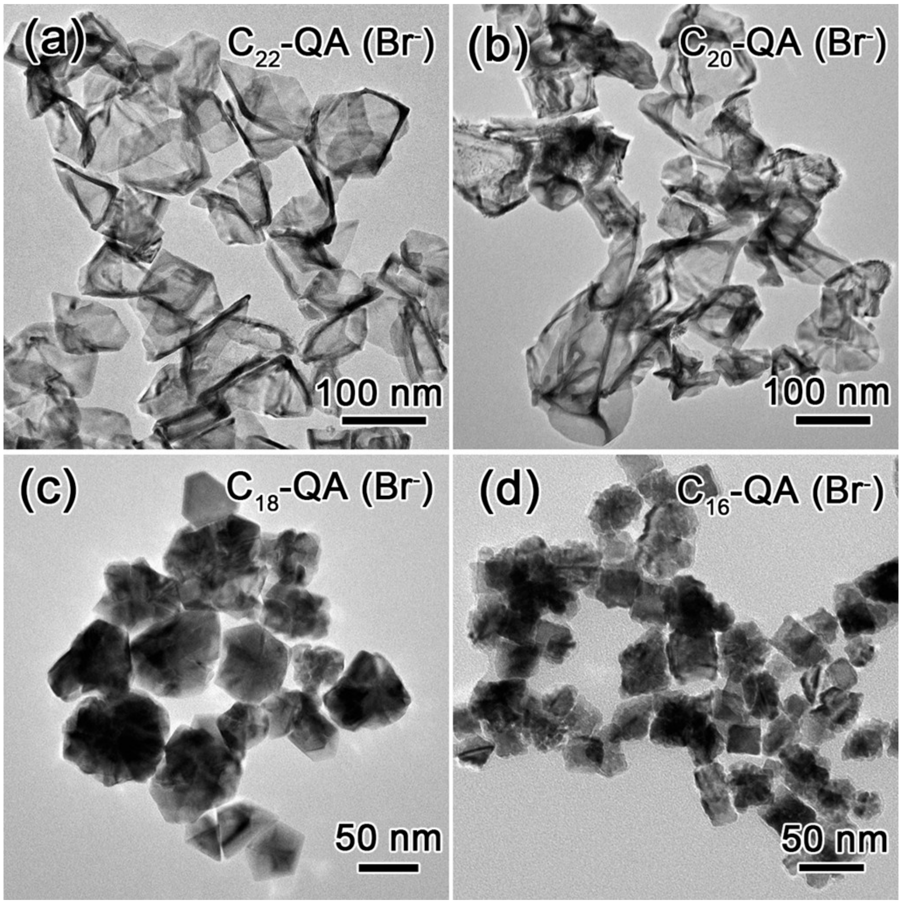

Fig. S15 Representative TEM images of Pd nanostructures synthesized using conventional QA-functionalized surfactants with different alkyl length: (a) C_{22}-QA (Br^-), (b) C_{20}-QA (Br^-), (c) C_{18}-QA (Br^-), and (d) C_{16}-QA (Br^-).

TEM images of Pd nanostructures obtained using C_{22}-QA (Br^-) and C_{20}-QA (Br^-) showed the successful formation of PdNSs although the thickness of nanosheets was slightly larger. Only bulk Pd crystals, however, were obtained using C_{18}-QA (Br^-) or C_{16}-QA (Br^-), indicating the importance of alkyl length on the synthesis of ultrathin PdNSs.

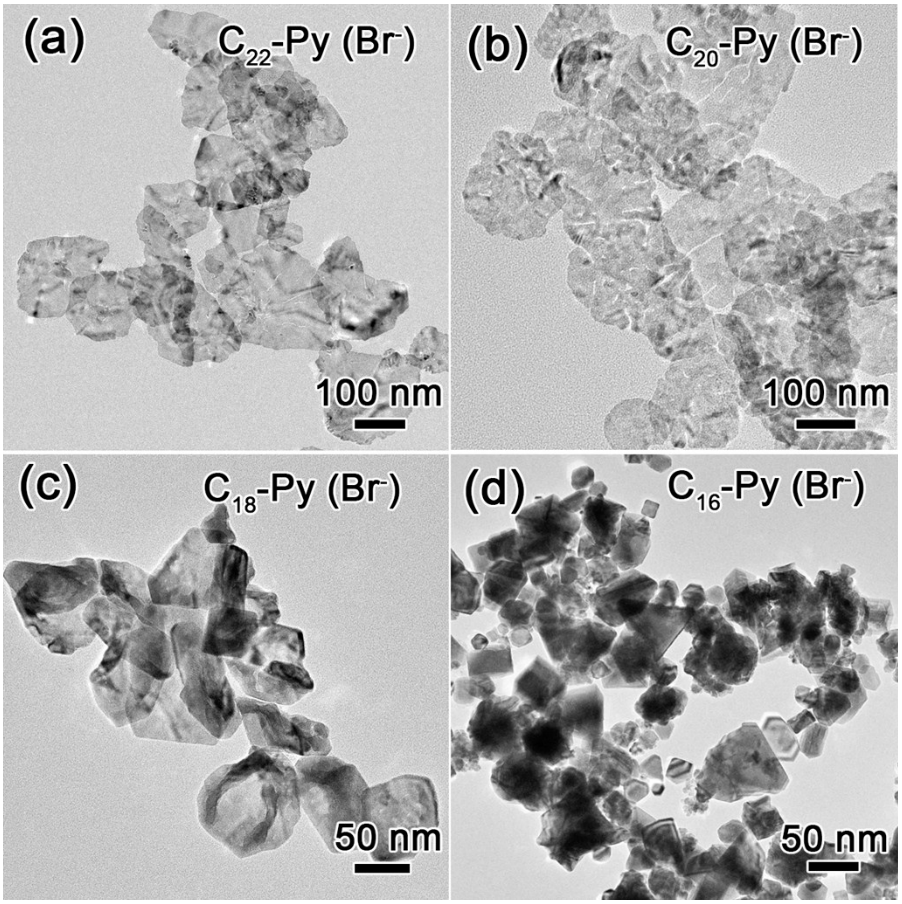

Fig. S16 Representative TEM images of Pd nanostructures synthesized using the Py-functionalized surfactants with different alkyl length: (a) C_{22}-Py (Br^-), (b) C_{20}-Py (Br^-), (c) C_{18}-Py (Br^-), and (d) C_{16}-Py (Br^-).

TEM images showed that Pd nanostructures obtained using C_{22}-Py (Br^-) and C_{20}-Py (Br^-) possessed the nanosheet morphology, while C_{18}-Py (Br^-) directed the synthesis of Pd nanoplates with the thicker height. By contrast, only bulk Pd nanoparticles could be synthesized using C_{16}-Py (Br^-) as the templates.

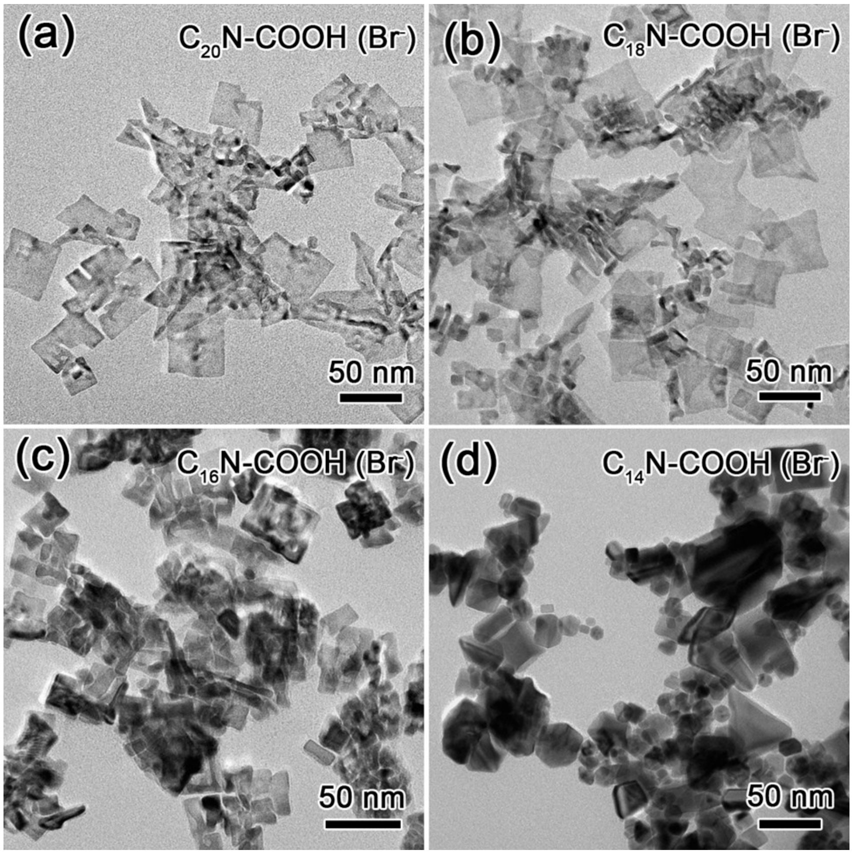

Fig. S17 Representative TEM images of Pd nanostructures synthesized using the COOH-functionalized surfactants with different alkyl length: (a) C_{20}N-COOH (Br^-), (b) C_{18}N-COOH (Br^-), (c) C_{16}N-COOH (Br^-), and (d) C_{14}N-COOH (Br^-).

Well-defined PdNSs were still obtained using the surfactants of C_{20}N-COOH (Br^-) and C_{18}N-COOH (Br^-). Pd nanoplates were formed using C_{16}N-COOH (Br^-), while bulk Pd crystals with irregular morphology were synthesized using C_{14}N-COOH (Br^-) or the surfactants with shorter alkyl chains. C_{18}N-COOH (Br^-) could direct the formation of PdNSs, in comparison to nanoplates and bulk nanocrystals synthesized using C_{18}-QA (Br^-) or C_{18}-Py (Br^-). The results further testified that the carboxyl groups were powerful for the confined growth of 2D PdNSs, due to the strong chemisorption interactions between Pd and oxygen atoms in carboxyl groups.

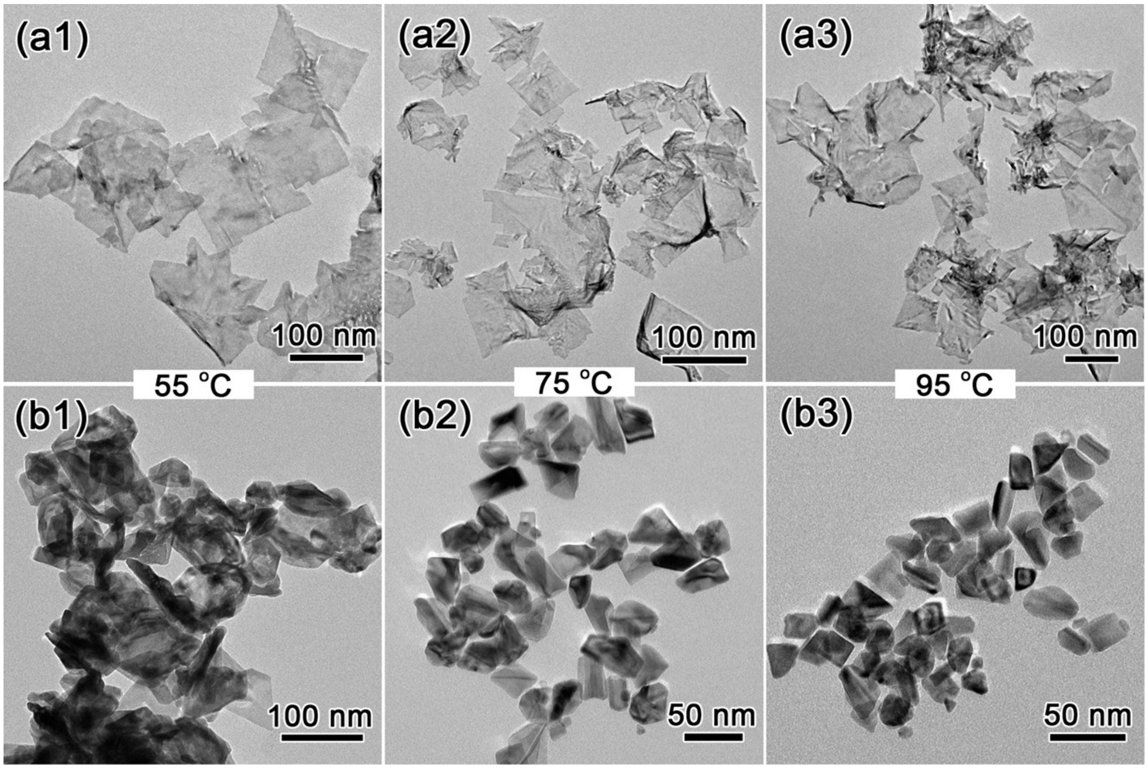

Fig. S18 TEM images of Pd nanostructures synthesized at different reaction temperature (55 °C, 75 °C, and 95 °C) using the surfactants of C_{22}N-COOH (Br^-) (a1-a3) and C_{22}-QA (Br^-) (b1-b3), respectively.

Using C_{22}N-COOH (Br^-) as the surfactant, ultrathin PdNSs could be obtained at the temperature range from 55 to 95 °C. However, even under 55 °C, only few Pd plates were produced using C_{22}-QA (Br^-). Bulk Pd nanoparticles formed at the higher temperature (above 55 °C). The results indicate the weaker chemisorption interaction between C_{22}-QA (Br^-) and Pd precursor/nanocrystals.

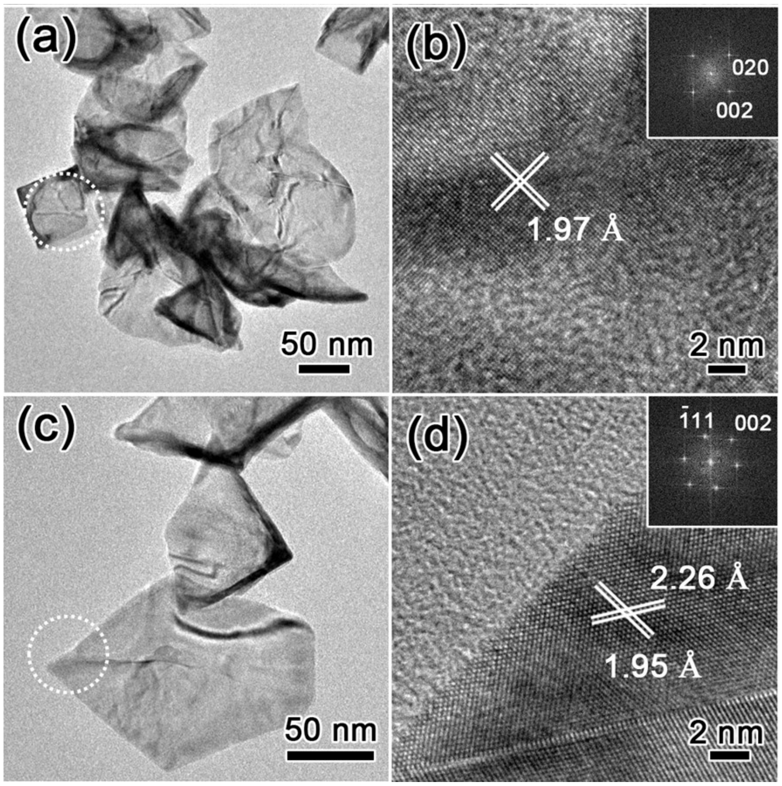

Fig. S19 (a, c) TEM, (b, d) high-resolution TEM images and corresponded FT patterns (inserted) of the PdNSs synthesized using the surfactant of C_{22}-QA (Br^-). Image b was taken along the [100] axes, while image d was taken along [110] axes. Two kinds of crystalline facets, {100} and {110}, were observed, indicating that C_{22}-QA (Br^-) cannot direct the construction of the PdNSs with single exposed facets. In comparison to Cl^- ion, Br^- has the selective and strong adsorption onto Pd {100} or {110} facets, resulting in the Pd crystals mainly enclosed by {100}/{110} facets.

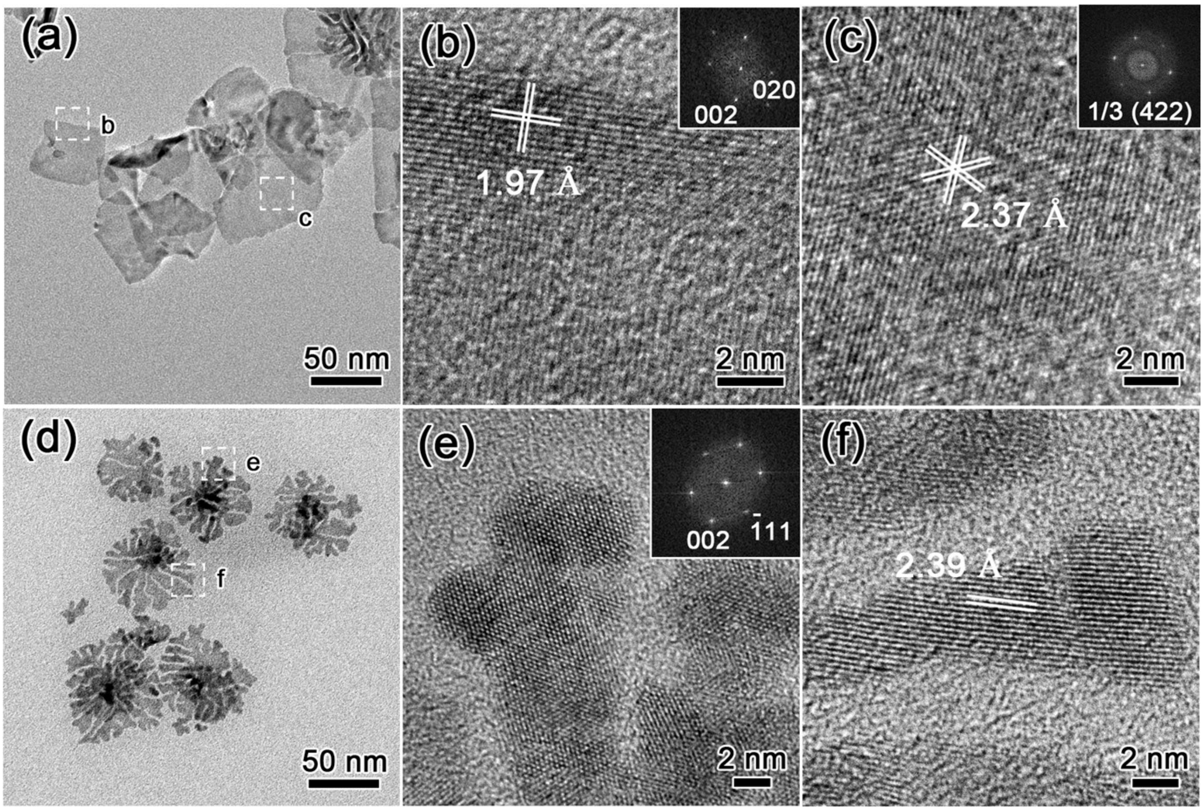

Fig. S20 (a, d) TEM and (b, c, e, f) high-resolution TEM images of the PdNSs synthesized using the surfactant of (a-c) C_{22}N-COOH (Cl^-) and (d-f) C_{22}-Py (Cl^-). The inserts in (b), (c) and (e) are the corresponded FT patterns. The fringes with a lattice spacing 2.37 Å or 2.39 Å was indexed to the Pd 1/3 (422) planes, while the fringe of 1.97 Å was indexed to the Pd (200) planes. Images b, c, e and f were taken along the {100}, {111}, {110} and {111} axes, respectively. The results indicated that the PdNSs synthesized using C_{22}N-COOH (Cl^-) composed of {100}/{111}-exposed mixed facets, while the exposed facets of PdNSs synthesized using C_{22}-Py (Cl^-) were {110} and {111}.

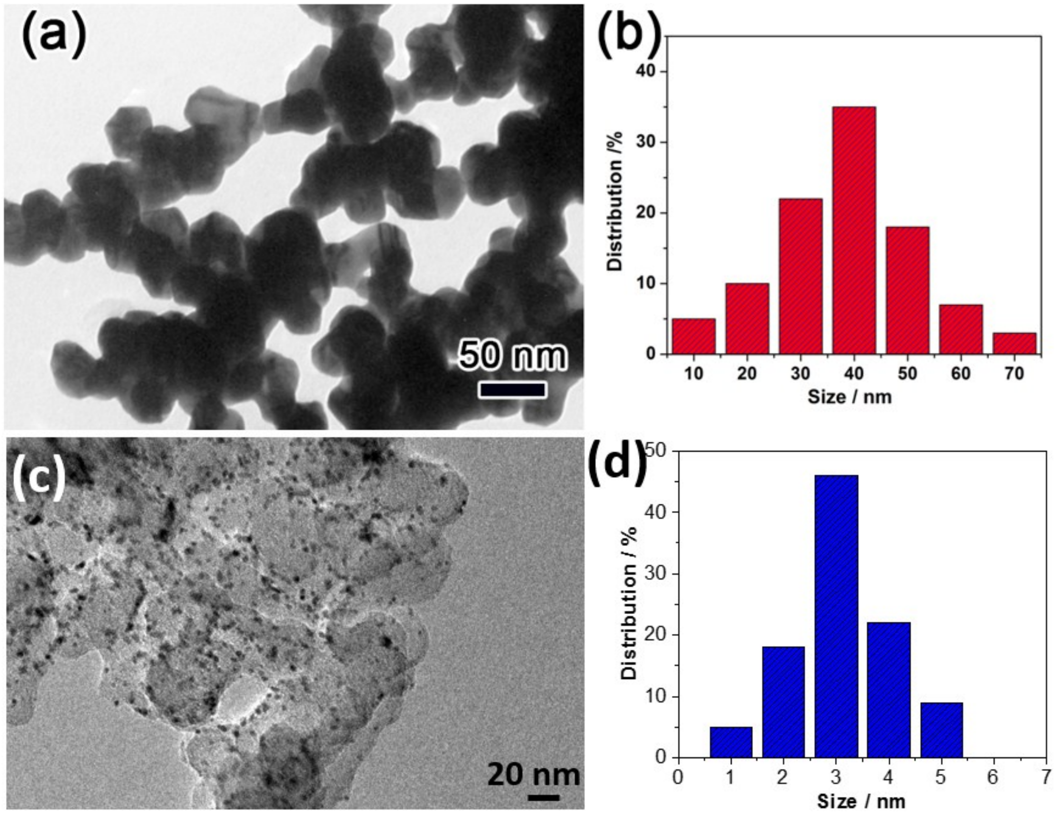

Fig. S21 (a, c) TEM images and (b, d) the corresponding size distributions of (a, b) PdB and (c, d) cPt used in the electrocatalytic tests.

Table S1. The summarization of all the sample information and corresponding synthetic conditions.

Surfactants	Reducing Agents	Synthesis Temperatures	Nanostructures	Exposed Facets	Sample Positions
**C_{22}N-COOH (Br^-)**	AA	35 ℃	Nanosheets	{100}	Figs. 2, S6-7, S9-10
**C_{22}N-COOH (Cl^-)**	AA	35 ℃	Nanosheets	{100} & {111}	Fig. S20a
**C_{22}-py (Br^-)**	AA	35 ℃	Nanosheets	{110}	Figs. 3a, S14
**C_{22}-py (Cl^-)**	AA	35 ℃	Dendritic Nanosheets	{110} & {111}	Fig. S20d
**C_{22}-QA (Br^-)**	AA	0 ℃	Nanosheets	{110} & {100}	Fig. S19
**C_{22}-QA (Cl^-)**	AA	0 ℃	Nanosheets	{111}	Fig. 3d
**C_{22}N-COOH (Br^-)**	CO	35 ℃	Nanosheets	{111}	Fig. S12a
**C_{20}N-COOH (Br^-)**	AA	35 ℃	Nanosheets	-*	Fig. S17a
**C_{18}N-COOH (Br^-)**	AA	35 ℃	Nanosheets	-	Fig. S17b
**C_{16}N-COOH (Br^-)**	AA	35 ℃	Nanoplate	-	Fig. S17c
**C_{14}N-COOH (Br^-)**	AA	35 ℃	Bulk Nanoparticles	-	Fig. S17d
**C_{20}-py (Br^-)**	AA	35 ℃	Nanosheets	-	Fig. S16b
**C_{18}-py (Br^-)**	AA	35 ℃	Nanoplate	-	Fig. S16c
**C_{16}-py (Br^-)**	AA	35 ℃	Bulk Nanoparticles	-	Fig. S16d
**C_{20}-QA (Br^-)**	AA	0 ℃	Nanosheets	-	Fig. S15b
**C_{18}-QA (Br^-)**	AA	0 ℃	Bulk Nanoparticles	-	Fig. S15c
**C_{16}-QA (Br^-)**	AA	0 ℃	Bulk Nanoparticles	-	Fig. S15d

***Do not test**
